# A Prime-Boost Immunization Strategy with Vaccinia Virus Expressing Novel gp120 Envelope Glycoprotein from a CRF02_AG Isolate Elicits Cross-Clade Tier 2 HIV-1 Neutralizing Antibodies

**DOI:** 10.3390/vaccines8020171

**Published:** 2020-04-07

**Authors:** Rita Calado, Joana Duarte, Pedro Borrego, José Maria Marcelino, Inês Bártolo, Francisco Martin, Inês Figueiredo, Silvia Almeida, Luís Graça, Jorge Vítor, Frederico Aires da Silva, Inês Dias, Belmira Carrapiço, Nuno Taveira

**Affiliations:** 1 Instituto de Investigação do Medicamento (iMed.ULisboa), Faculdade de Farmácia, Universidade de Lisboa, 1649-003 Lisboa, Portugal; ritadiogoalmeida@gmail.com (R.C.); joanadnduarte@gmail.com (J.D.); pborrego65@gmail.com (P.B.); josemarcelino@ff.ulisboa.pt (J.M.M.); ibartolo@ff.ul.pt (I.B.); rickmartin_5@hotmail.com (F.M.); inesb.figueiredo@hotmail.com (I.F.); 2 Centro de Investigação Interdisciplinar Egas Moniz (CiiEM), Instituto Universitário Egas Moniz, 2829-511 Monte de Caparica, Portugal; 3 Faculdade de Medicina, Instituto de Medicina Molecular, Universidade de Lisboa, 1649-02 Lisboa, Portugal; scpalmeida@gmail.com (S.A.); lgraca@fm.ul.pt (L.G.); 4 Post-Graduate Program in Infectious Diseases, and Department of Social Medicine, Center of Health Sciences, Federal University of Espirito Santo, Vitória 29075-910, Brazil; 5 Biochemistry and Human Biology Dept, Faculdade de Farmácia, Universidade de Lisboa, 1649-003 Lisboa, Portugal; jvitor@netcabo.pt; 6 Faculdade de Medicina Veterinária, Universidade de Lisboa, 1300-477 Lisboa, Portugal; fasilva@fmv.ulisboa.pt (F.A.d.S.); inocas@fmv.ulisboa.pt (I.D.); belmira@fmv.ulisboa.pt (B.C.)

**Keywords:** HIV-1 vaccine, Recombinant Vaccinia virus, envelope glycoproteins, non-B-non-C clades, BALB/c mice, New Zealand White rabbits, broadly neutralizing antibodies

## Abstract

Development of new immunogens eliciting broadly neutralizing antibodies (bNAbs) is a main priority for the HIV-1 vaccine field. Envelope glycoproteins from non-B-non-C HIV-1clades have not been fully explored as components of a vaccine. We produced Vaccinia viruses expressing a truncated version of gp120 (gp120t) from HIV-1 clades CRF02_AG, H, J, B, and C and examined their immunogenicity in mice and rabbits. Mice primed with the recombinant Vaccinia viruses and boosted with the homologous gp120t or C2V3C3 polypeptides developed antibodies that bind potently to homologous and heterologous envelope glycoproteins. Notably, a subset of mice immunized with the CRF02_AG-based envelope immunogens developed a cross-reactive neutralizing response against tier 2 HIV-1 Env-pseudoviruses and primary isolates. Rabbits vaccinated with the CRF02_AG-based envelope immunogens also generated potent binding antibodies, and one animal elicited antibodies that neutralized almost all (13 of 16, 81.3%) tier 2 HIV-1 isolates tested. Overall, the results suggest that the novel CRF02_AG-based envelope immunogens and prime-boost immunization strategy elicit the type of immune responses required for a preventive HIV-1 vaccine.

## 1. Introduction

Developing a safe, effective, and affordable vaccine to prevent HIV infection is the best hope for controlling or ending the HIV epidemic. The search for a preventive vaccine faces enormous challenges, namely (i) the absence of well-defined immune correlates of protection against HIV in humans, (ii) uncertainty about the best animal model to predict human responses to vaccines, (iii) the failure in the induction of broadly neutralizing antibodies (bNAbs) by different antigens, and (iv) the extraordinary sequence diversity of HIV-1 and its capacity to constantly mutate, evolve, and escape from the host immune response [1,2,3,4,5,6]. There are at least nine HIV-1 genetic subtypes as well as multiple recombinant forms worldwide. Five HIV-1 strains dominate the global epidemic: C (50%), A (12%), and B (11%), followed by CRF02_AG (8%), G (5%), and CRF01_AE (5%) [7]. Other subtypes, like J and H, represent less than 1% of infections. An ideal vaccine immunogen should be able to contend with the remarkably high diversity of HIV-1 and induce an immune response able to cross-react with contemporaneous heterologous viruses. 

Although correlates of protection from HIV-1 infection are not completely defined, there are several studies that support the crucial role of neutralizing antibodies in preventing HIV-1 infection [4,6,8]. In some individuals, broadly neutralizing antibodies (bNAbs) emerge after a few years of infection, and these antibodies are able to neutralize a diverse range of viruses, including tier 2 virus, that dominate human transmissions or even tier 3 viruses with a higher resistance profile [4,9,10]. Passive immunization studies in animal models have demonstrated that administration of some bNAbs can protect from infection [11]. 

Neutralizing epitopes on HIV-1 include the CD4 binding site, V1/V2 loops, V3 loop, gp120/gp41 interface region, and the fusion peptide and MPER (Membrane-proximal external region) in gp41 [12,13,14,15,16,17,18,19,20]. In contrast with the CD4 binding site, which is highly conserved, V1, V2, and V3 are variable regions. V3 is the most conserved region of the three variable regions, and it harbors a highly conserved motif, GPGR/Q (residues 312–315 in the HXB2) [21]. V3 is a highly immunogenic region and anti-V3 monoclonal antibodies such as 447-52D neutralize up to 50% of the viruses in various multiclade panels [13,14,16,17,21,22]. 

Despite the urgent need for a vaccine, only six HIV-1 vaccine candidates have completed efficacy trials. The prime-boost regimen used in the RV144 trial is still the only immunization strategy that has demonstrated some level of protection against HIV-1 infection [23,24]. The immunization strategy of this trial consisted of four priming injections of an attenuated recombinant canarypox vector vaccine (ALVAC/vCP1521) expressing env, gag, and protease genes and two booster injections of a B/E recombinant glycoprotein gp120 subunit (AIDSVAXB/E). Immune responses observed in the RV144 trial that were associated with a reduced risk of HIV-1 infection included non-neutralizing antibodies to V1/V2, high levels of antibody-dependent cellular cytotoxicity (ADCC) after controlling for IgA, and HIV-1-specific IgG3 responses [23,24,25]. RV144 recipients developed low titers of neutralizing antibodies that were only active against tier 1 isolates likely explaining the modest results obtained in this trial [26]. Similar results were obtained in the HVTN 097 clinical trial that was conducted in South Africa using the same immunogens and vaccination strategy of RV144 [27]. Unfortunately, HVTN 702, a phase 2b/3 HIV vaccine trial involving a clade-C version of the immunogens used in RV144 was halted recently due to lack of protection. 

Since bNAbs are considered the best correlate of protection against HIV infection, the development of envelope immunogens that elicit bNAbs against tier 2 and tier 3 HIV-1 isolates is currently the main priority for the HIV-1 vaccine field [4,10,26]. So far, no vaccine candidate has been able to consistently induce bNAbs against heterologous tier 2 and 3 isolates from different clades. The majority of HIV vaccine regimens consists of prime-boost regimens that use different combinations of immunogens, namely, recombinant virus expressing HIV envelope [28,29,30], or/and plasmid DNA constructs [31], or/and various HIV purified proteins (e.g., native-like trimers) or peptides [3,29,32,33,34,35,36,37]. Recent studies in mice, rabbits, and non-human primates have shown that it is possible to elict NAbs against tier 2 HIV-1 isolates, although sporadically, with limited breadth and at low levels [3,20,29,33,36,38,39]. In contrast, in cows, immunization with the trimer envelope glycoprotein BG505-SOSIP.664 (clade A) resulted in the rapid elicitation of broad and potent serum antibody responses in all immunized animals [40]. Notwithstanding these recent successes, the induction of efficient and consistent bNAb responses against heterologous tier 2 and tier 3 viruses from different clades has been a difficult task for the majority of vaccine candidates.

In the present study, we aimed to contribute new envelope-based immunogens derived from non-B clades for inclusion in a HIV-1 vaccine fit for old and very diverse HIV-1 epidemics such as those of Angola and other countries in Central Africa [41]. We produced a new set of recombinant Vaccinia virus vectors expressing a truncated form of gp120 from several primary isolates of HIV-1 of non-B clades mostly coming from Angola. We also produced soluble gp120 glycoproteins and polypeptides comprising the C2V3C3 envelope regions of the same isolates. Using a prime-boost vaccination regimen, we were able to elicit cross-reactive gp120-binding antibodies in mice and rabbits and, in some animals, antibodies that neutralized heterologous tier 2 HIV-1 isolates from different clades. 

## 2. Materials and Methods 

### 2.1. Ethics Statement

BALB/cByJ (H-2d) mice were maintained under specific pathogen-free conditions at the Instituto de Higiene e Medicina Tropical and Instituto de Medicina Molecular, where all animal work was performed in accordance with Directive 2010/63/EU. Experimental animals were females between 8–10 weeks. Eight female New Zealand White rabbits were purchased and maintained at the Faculdade de Medicina Veterinária, Lisboa (FMV), Portugal. Permission for animal experimentation was granted by ORBEA-iMM (the institutional Animal Welfare Body), Ethics and Animal Welfare Committee (CEBEA) of the Faculty of Veterinary Medicine, Lisbon, and DGAV (Portuguese competent authority for animal protection) under the numbers 022870/2016 and CEBEA/PGG/054/2015. 

### 2.2. Cells, Plasmids, Viruses, and Antibodies

Rat2 (TK-) cells were purchased from American Type Culture Collection (Rockville, MD, USA). HeLa cells (ATCC® CCL-2™) were obtained from American Type Culture Collection. TZM-bl cells were provided by the AIDS Research and Reference Reagent Program (ARRRP), National Institutes of Health. HeLa, Rat2, and TZM-bl cells were cultured in complete growth medium that consists of Dulbecco´s minimal essential medium (DMEM) supplemented with 10% v/v fetal bovine serum (FBS), 100 U/mL of penicillin-streptomycin (Gibco/Invitrogen, Waltham, MA, USA), 1mM of sodium pyruvate (Gibco/Invitrogen, Waltham, MA, USA), 1mM of L-glutamine (Gibco/Invitrogen, Waltham, MA, USA), and 1mM of non-essential amino acids (Gibco/Invitrogen, Waltham, MA, USA). All cell cultures were maintained at 37 ˚C in 5% CO_2_. The following items were also obtained from the ARRRP: Western Reserve Strain of Vaccinia virus (VV_WR_), a Panel of Global tier 2 HIV-1 Env Clones (cat#12670) designed to assess neutralization responses, HuMAbs 447-52D (anti-V3), VRC01 (anti-CD4bs), 3BNC117 (anti-CD4bs), HJ16 (anti-CD4bs), PG16 (anti-V2), 2G12 (anti-N-linked glycans in C2, C3, V4, C4), b12 (anti-CD4bs), and 2F5 (anti-MPER), recombinant proteins M.CON.SD11gp120 and SF162 gp140 Trimer.

### 2.3. Cloning of Envelope Genes from Primary Isolates 

Envelope genes expressed in this study were obtained by PCR amplification from primary isolates derived from HIV-1 infected individuals from Angola and Portugal (Table S1). Viral genomic RNA was extracted and reverse transcription (RT) was performed using Titan One Tube RT-PCR System (Roche Diagnostic Systems). Full-length env genes were amplified by nested PCR as described previously [42], purified using JETQUICK Gel Extraction Spin Kit (GENPRICE, Yurok Circle San Jose, CA, USA), and sequenced. Primers used for amplification and sequencing are described in Tables S2 and S3. For subtyping, the nucleotide sequences were aligned with reference sequences using ClustalX4 (http://www.clustal.org/clustal2/). The find model tool (https://www.hiv.lanl.gov/content/sequence/findmodel/findmodel.html) was used to estimate the best nucleotide substitution model, and the PhyML v3.1 program in SeaView was used to compute maximum likelihood phylogenetic trees [43]. 

CCR5 and/or CXCR4 use of primary isolates was determined in TZM-bl cells (CD4+, CCR5+, CXCR4+) in the presence of excessive amounts of the CCR5 antagonist TAK-779 (10µM) and/or of the CXCR4 antagonist AMD3100 (1.2µM) as previously described [44]. For envelope genes derived from plasma, coreceptor usage was determined based on the sequence of V3 using the geno2pheno algorithm [45]. For each subtype, a group of newly selected primers was designed based on the env sequences to amplify the gp120 region lacking 78 bases at the carboxyl terminus of the C5 region (therefore named truncated gp120 or gp120t). Forward and reverse primers included a restriction site for Sal I (5´- GTCGAC-3´); reverse primers also included a stop codon (CTA) before the restriction site. PCR primer numbers and positions are described in Table S2. All amplifications were performed using the Expand Long Template PCR system (Roche Diagnostic Systems) according to the manufacturer’s instructions. Amplified DNA was cloned into the Sal I site of the Vaccinia virus insertion vector pMJ601. In this vector, protein expression is driven by the strong late Vaccinia virus promoter present in pMJ601 [46].

### 2.4. Production of Recombinant Vaccinia Viruses 

Recombinant Vaccinia viruses expressing the truncated gp120 glycoproteins (gp120t) were obtained as described [30]. Briefly, HeLa cells were transfected with recombinant pMJ601 plasmids using jetPRIME® (Polyplus transfection SA, New York, NY, USA) according to the manufacturer´s instructions, and cells simultaneously infected with Vaccinia virus strain VV_WR_ and recombinant Vaccinia viruses resistant to 5-bromodeoxyuridine and expressing β-galactosidase were selected in Rat2 cells. The recombinant viruses were titrated using the method of Reed and Muench [47].

### 2.5. Expression and Purification of Truncated gp120 Env Glycoproteins 

The method of Rose et al. [48] was used to produce soluble truncated gp120s (Sgp120t). Briefly, HeLa cells were infected with 5 PFU of recombinant Vaccinia virus per cell and incubated for 3 h. Medium containing the infecting virus was replaced with DMEM supplemented with 2.5% v/v FBS at 3 h post infection. After 24 h of infection, medium containing Sgp120t was collected, clarified by centrifugation at 3000 xg for 10 min and filtered with a 0.2 µM pore size filter to remove Vaccinia virus. Cells were washed with cold phosphate buffered saline (PBS) and lysed using RIPA + DOC buffer (0.15M NaCl, 0.05M Tris-HCl, 1% Triton X-100, 1% DOC, 0.1% SDS). Lysates were centrifuged at 35.000 rpm for 60 min at 4 °C and the supernatant containing the proteins was collected. 

Purification of Sgp120t-AG, the soluble truncated gp120 from the CRF02_AG isolate, from cell culture supernatant was performed using lectin affinity chromatography as described [49]. Agarose-bound Galanthus nivalis lectin (GNL) (Vector Laboratories, Burlingame, CA, USA) was used which binds to the terminal D-mannose groups of HIV gp120. For purification, 1000 mL of HeLa cell culture supernatant containing Sgp120t-AG in complete DMEM medium without FBS was used. Briefly, a Sigma 1.0 × 10 cm chromatography column was filled with 4 mL of GNL slurry, settled, and washed with 40 mL of PBS at 0.2mL/min. Then cell culture supernatant was pumped through the lectin column at 1 mL/min, and the flow-through was collected to determine the efficiency of the binding. After washing with ten volumes of cold PBS, 5 mL of a mannose solution (0.5 M methyl alpha-D-manno-pyranoside, Sigma, Lisbon, Portugal) was added at 0.25 mL/min. Eluted proteins were collected with a Frac-100 collector (Pharmacia) in 2 mL fractions and absorption at 280 nm was measured (Nanodrop UV spectrophotometer). Finally, contaminating proteins from the eluted fractions were removed through metal-affinity chromatography using TALON Superflow (GE) charged previously with 200 mM cobalt chloride (2 mL on a Sigma 1.0 × 10 cm chromatography column). Flow-through fractions containing Sgp120t-AG were collected and concentrated using Vivaspin 6® >100.000 MWCO PES according with the manufacturer´s instructions. Proteins obtained were analyzed on 7.5% SDS-PAGE followed by BlueSafe protein stain (Nzytech, Lisbon, Portugal) and quantified using Nanodrop. The gel was scanned using Kodak 1D image analysis software, and the purity of the monomeric form of gp120t_AG was estimated to be 82.5%.

### 2.6. Antigenic Characterization of Envelope Glycoproteins

Antigenic reactivity of envelope glycoproteins was quantified by ELISA and Western blot (WB). For WB analysis, proteins separated by 7.5% SDS-PAGE under non-reducing conditions (i.e., without β-mercaptoethanol) were transferred to nitrocellulose membranes 0.45 µM (BioRad, Hercules, CA, USA) treated with blocking buffer (1 × TBSt with 4% w/v nonfat dry milk) and incubated with serum from HIV-1 and HIV-2 infected individuals diluted (1:200) in primary antibody buffer (1X TBSt with 4% w/v nonfat dry milk and 5% goat serum) and/or human monoclonal antibodies 447-52D (anti-V3), VRC01 (anti-CD4bs), 3BNC117 (anti-CD4bs), HJ16 (anti-CD4bs), PG16 (anti-V2), 2G12 (anti-N-linked glycans in C2, C3, V4, C4), b12 (anti-CD4bs), and 2F5 (anti-MPER) at 1 µg/mL. Next, the membrane was washed with TBSt 0.25% and incubated with anti-human IgG- alkaline phosphatase antibody produced in goat (Sigma) and/or anti-human IgG-peroxidase antibody produced in goat (Sigma, Lisbon, Portugal). Colorimetric detection of proteins was performed by AP conjugate Substrate Kit (BioRad, Hercules, CA, USA), and chemiluminescent detection was performed with Pierce™ ECL Western Blotting Substrate (ThermoFisher Scientific, Waltham, MA, USA). Quantification of glycoproteins was done using Kodak 1D image analysis software.

Binding specificity of Sgp120 t glycoproteins was analyzed in ELISA assays against the eight human monoclonal antibodies described above at a final concentration of 10 µg/mL. For this purpose, Immuno MaxiSorp 96-well microplates (Nunc) were coated with the Sgp120t glycoproteins (~2.3–4 µg per well) in 0.05M bicarbonate buffer. Envelope gp120 of HTLV-IIIB, clone BH8 (expressed by recombinant Vaccinia virus vPE16), SF162 gp140 (2 µg/mL), and M.CON.S D11 gp120 (2 µg/mL) were used as positive controls. HIV-1 plasma samples at a final dilution of 1:200 were used as positive antibody control, and 2F5 antibody (anti-gp41) was used as a negative control. Except for SF162 gp140, the cut-off value of the assay was calculated as the mean OD value of 2F5 reactivity plus 2 times the SD. The cut-off value of the assay with SF162 gp140 (which reacts with 2F5) was calculated as the mean OD value of HIV-seronegative samples plus twice the SD.

### 2.7. Production, Expression, and Analysis of C2V3C3 Polypeptides 

A DNA fragment of 534 nucleotides comprising the C2, V3, and C3 coding regions from all isolates (position 6858–7392 in HXB2) was amplified using primers described in Table S2 and cloned into the bacterial expression vector pTrcHis (Invitrogen). Expression of C2V3C3 polypeptides with 178 amino acids (position 212–390 in gp120 in HXB2) in *Escherichia coli* strain TOP10 was induced with isopropyl-β-D-thiogalactopyranoside (IPTG) according to the manufacturer´s instructions. Protein purification was performed using Dynabeads® His-tag Isolation & Pulldown (Life Technologies). Bradford assay (Bio-Rad) was performed to determine protein concentration. Purified recombinant polypeptides were analyzed by SDS-12% PAGE, and the antigenic structure of the purified polypeptides was analyzed by an ELISA assay with plasma samples from HIV-1 infected patients from Angola (*n* = 28). Briefly, Immuno MaxiSorp 96-well microplates (Nunc) were coated with recombinant polypeptides from HIV-1 subtypes C, CRF02_AG, J, G, and H (2.5 µg/mL) diluted in 0.05M bicarbonate buffer (pH 9.4) by overnight incubation at 4 °C and blocked with 2% gelatin (Bio-Rad). Plasma from HIV-1 infected patients (starting at 1:100) was added to the microplates and, after 2 h of incubation at room temperature, anti-human IgG (Fc specific)-alkaline phosphatase antibody produced in goat (Sigma) was added as a secondary antibody. Colorimetric reaction was developed with SIGMAFAST™ p-Nitrophenyl phosphate (pNPP) tablets and read at 405 and 492 nm against a reference wavelength of 620 nm on a microplate reader. The clinical cut-off value of the assay, calculated as the mean OD value of HIV-seronegative samples plus twice the SD, was determined using samples from healthy HIV-seronegative subjects. Binding antibody titer was calculated as the highest plasma dilution giving a positive reaction (OD/cut-off > 1). 

### 2.8. BALB/c Mice Immunizations

Recombinant Vaccinia viruses expressing gp120t (VVgp120t), soluble filtered truncated gp120 (Sgp120t), and recombinant polypeptides C2V3C3 described above were used as vaccine immunogens in two separate immunization experiments. In the first experiment, the pilot study, we used the immunization strategy that was proven efficient in our previous HIV-2 vaccination experiment [30], with some minor modifications. A total of 20 six-week-old BALB/c mice divided into six groups (Groups 1–6, mice 1–20) were first immunized intraperitoneally (IP) with 2 × 10^7^ PFU of recombinant Vaccinia viruses expressing gp120t from HIV-1 clades C, CRF02_AG, and J (Table 1). Mice were boosted (IP route) at days 15 and 30 with 10 µg of the homologous C2V3C3 polypeptide and at day 45 with the homologous Sgp120t (10 µg). Groups 1 and 2 were control groups. Group 1 received no immunogen and Group 2 was immunized with VV_WR_. Three to four mice were used in each group with the exception of Group 1, which included only two mice. Of note, although Groups 3 and 4 received exactly the same immunogens, C2V3C3 polypeptide administered to Group 4 was eluted with urea instead of imidazole. 

In the second experiment, the main study, thirty 8- to 10-week-old female BALB/c ByJ (H-2d, BALB/c) were included. Animals were divided into 11 groups according to the immunogen clade and immunization strategy (Table 2). Animals from Groups 3, 4, and 5 were primed (IP route) with 2 × 10^7^ PFU of recombinant Vaccinia virus expressing gp120t and boosted twice with recombinant polypeptides C2V3C3 (10µg) and once with Sgp120t (10 µg). Animals from Groups 3A, 4A, and 5A were primed with recombinant Vaccinia virus expressing gp120 and boosted three times with Sgp120t. Finally, animals from Groups 3B, 4B, and 5B were primed with Sgp120t and boosted twice with recombinant polypeptides C2V3C3 and once with Sgp120t. Groups 1 and 2 were control groups. Group 1 did not receive any immunogen, and Group 2 was immunized with VV_WR_. Complete Freund’s adjuvant (CFA) (Sigma) was used for priming in Groups 3B, 4B, and 5B, whereas Incomplete Freund’s adjuvant (IFA) (Sigma) was used for all the boosts. 

In both studies, the schedule of immunization consisted of one priming at day 1 and three boosts at days 15, 30, and 45. Mice sera were collected immediately before each immunization and 15 days after the last immunization. Mice were sacrificed on day 60.

Rabbits were divided into three groups: Group 1 (placebo) was immunized with 500 µL of PBS; Group 2 (control) was primed with 500 µL of 2 × 10^7^ PFU of Vaccinia virus strain WR (VV_WR_) and boosted three times with 500 µL of supernatant of cells infected with VV_WR_ (S_WR_); and Group 3 was primed with 500 µL of 2 × 10^7^ PFU of recombinant VVgp120t-AG and boosted three or four times with 500 µL of Sgp120t-AG (corresponding to 35 µg of glycoprotein) (Table 3). For all groups, boosts were performed at days 35, 63, and 121 (boost I, II, and III). Two rabbits of Group 3 (R6 and R8) received an extra boost at day 150 (boost IV). S_WR_ and Sgp120t-AG boosts were emulsified in IFA in a 1:1 ratio. All immunizations were administered subcutaneously, and blood samples were collected from the marginal ear vein. Rabbit sera were collected immediately before each immunization plus twice between boost II and III to access for binding antibodies. All rabbits were sacrificed on day 154. 

### 2.9. Envelope-specific Antibody Binding Reactivity in Sera of Immunized Animals

To analyze serum antibody responses in mice, Immuno MaxiSorp 96-well microplates (Nunc) were coated with Sgp120t (clades B, C, AG and J) and recombinant polypeptides C2V3C3 (clades C, AG, and J) as described above. After overnight incubation at 4 °C, microplates were blocked with 2% gelatin (Bio-Rad). Mice anti-sera from days 0, 15, 30, 45, and 60 were heat-inactivated for 1 h at 56 °C, added to the microplates (1:200 final dilution in a total volume of 100 µL), and incubated for 2 h at room temperature. Anti-mouse IgG-alkaline phosphatase antibody produced in goat (Sigma) was added to the microplates as secondary antibody (1:2000 dilutions). Colorimetric reaction was developed as described above. Negative controls were pre-immune serum and serum from mice immunized with VV_WR_. Positive control was sera from HIV-1-infected individuals. In this case, the secondary antibody was anti-human IgG- alkaline phosphatase antibody produced in goat (Sigma). Sera with an optical density (OD) above the pre-immune controls were considered positive. 

In rabbits, HIV-1 gp120-specific binding antibodies were analyzed by ELISA using sera from six time points (T0-T5, Table 3). Briefly, Immuno MaxiSorp 96-well microplates (Nunc) were coated with 2.3 µg of autologous Sgp120t-AG and heterologous recombinant gp120 derived from a consensus HIV-1 group M envelope gene sequence (M.CON.SD11) at 1 µg/mL. Rabbit sera from the last time point (T5, day 154) were also tested against the heterologous gp140 trimer (SF162) at 1 µg/mL. All proteins were diluted in 0.05M bicarbonate buffer. After overnight incubation at 4 °C, microplates were blocked with 2% gelatin (Bio-Rad). Rabbit anti-sera (1:300 final dilution) from days 1, 35, 63, 99, 121, and 154 were heat-inactivated for 1 h at 56 °C [50], added to the microplates and incubated for 2 h at room temperature. Anti-rabbit IgG-alkaline phosphatase antibody produced in goat (Sigma) was added at 1:2000 dilutions to the microplates as a secondary antibody. Colorimetric reaction was developed with SIGMAFAST™ p-Nitrophenyl phosphate (pNPP) tablets and read at 405 and 492 nm against a reference wavelength of 620 nm on a microplate reader. Positive (serum from HIV-1-infected individuals) and negative (serum from healthy donors) controls were used at 1:200 final dilutions. Sera from pre-immunized rabbits and from rabbits immunized with VV_WR_ were used as additional negative controls of the assay. Sera with an optical density (OD) above the cut-off (mean OD of rabbits immunized with VV_WR_ immunogens plus four times the standard deviation) were considered positive. Binding antibody titer of rabbit sera from the last time point (T5) against autologous and heterologous gp120 glycoproteins was defined as the highest serum dilution (log_10_) at which a positive reaction (OD/cut-off > 1) was obtained. 

Reactivity of Sgp120t envelope glycoproteins against rabbit sera from the last time point was also analyzed by WB as described above. For this propose, rabbit sera were used at 1:200 final dilution in primary antibody buffer, and anti-rabbit IgG-alkaline phosphatase antibody produced in goat was used as a secondary antibody at 1:2000. Colorimetric detection of proteins was performed using AP conjugate Substrate Kit (BioRad).

### 2.10. Neutralization Assays

A panel of HIV-1 viruses including 12 tier 2 HIV-1 from a reference panel [51] (Table S4), five primary isolates (two subtypes J, two untypable U, and one CRF02_AG) (Table S1), and two tier 1 isolates (NL4.3 and SG3.1) were used in neutralizing assays. Primary isolates were obtained from HIV-1-infected patients by co-cultivation with peripheral blood mononuclear cells (PBMCs) from seronegative subjects. Env-pseudotyped viruses were produced by transfection of Env-expressing plasmids in 293T cells using pSG3.1Δenv as backbone and titrated in TZM-bl cells as described [51].

Neutralizing activity of mice sera was tested using a single-round viral infectivity assay using a luciferase reporter gene assay in TZM-bl cells, as described previously [30,52]. Briefly, the cells (10,000 cells in 100 µL of DMEM supplemented with 10% heat-inactivated FBS) were added to each well of 96-well flat-bottom culture plates (Nunc) and allowed to adhere overnight. Next, 100 µL of each virus (200 TCID50/well) were incubated for 1 h at 37 °C with heat-inactivated (56 °C, 1 h) mice sera in a total volume of 200 µL of growth medium (DMEM supplemented with 10% heat-inactivated FBS) containing DEAE-Dextran (19.7 µg/mL) and added to the cells. Final serum dilution used in the assays was 1:40. After 48 h, culture medium was removed from each well and plates were analyzed for luciferase activity on a luminometer (TECAN) using Pierce™ Firefly Luciferase Glow Assay Kit (ThermoFisher Scientific). Wells with medium were used as background control, and virus-only controls were included as infection control. The effect of pre-immune serum on infection was used as baseline neutralizing activity. To monitor the specificity of neutralization, each serum sample was also tested against pseudoviruses carrying the vesicular stomatitis virus (VSV) envelope and against two primary isolates of HIV-2. Sera from HIV-1-infected individuals and human monoclonal antibodies PG9 and VRC-CH31 at a final concentration ranging between 0.05 and 0.5 µg/mL were used as positive controls of the assay [51]. Percent neutralization of animal sera was determined by calculating the difference in average RLU (Relative Light Units) between test wells containing post-immune sera and test wells containing pre-immune sera after the normalization of the results using the average RLU of cell controls. Results were considered valid if the average RLU of virus control wells was ≥10 times the average RLU of cell control wells. Neutralization percentage of the positive controls was determined by calculating the difference in average RLU between test wells (cells + sample + virus) and cell control wells (cells only), dividing this result by the difference in average RLU between virus control (cell + virus) and cell control wells, subtracting from 1, and multiplying by 100 [53]. A positive neutralization was considered when at least 50% reduction in RLU values was observed relative to RLU values from virus control wells after subtraction of background RLU. Failure to score at least 50% reduction of RLU at any serum dilution was considered a negative result. Neutralization assays were performed with sera obtained 15 days after the last immunization (day 60). All mouse sera were tested for neutralizing antibodies once in triplicate at 1:40 dilution. The neutralization assays were repeated for animals that neutralized at least one tier 2 isolate at more than 50% in the first assay. 

Neutralizing activity in rabbits was also tested using a single-round viral infectivity assay using a luciferase reporter gene assay in TZM-bl cells, and percent neutralization was determined as described above. In this case, final serum dilution used was 1:20. Neutralizing antibody titers were determined for the rabbit (R8) showing neutralizing activity (>50%). In this case, 100 µL of 2-fold serial dilutions beginning at 1:20 were mixed with 100 µL of each virus and incubated for 1 h before adding to the cells. After 48 h, culture medium was removed from each well, and plates were analyzed for luciferase activity as described above. Wells with medium were used as background control, and virus-cell wells were included as infection control. The effect of pre-immunized serum on infection was used as baseline neutralization. Sera from HIV-1-infected individuals (1:40) and monoclonal antibodies PG16, HJ16, and VRC01 at a final concentration between 0.001 and 2 µg/mL were used as positive controls of the assay [54]. Neutralizing titer (ID50) was defined as the highest dilution for which 50% of neutralization was achieved. Neutralizing assays were performed with sera from the last timepoint (day 154). To monitor the specificity of neutralization, each serum sample was also tested against pseudoviruses carrying the vesicular stomatitis virus (VSV) envelope and against two primary isolates of HIV-2.

Baseline activity from neutralizing assays in mice and rabbits is shown in Table S5.

### 2.11. Statistical Analysis

Statistical analysis was performed with GraphPad Prism 5.0. C2V3C3-specific binding responses are summarized by the median and the interquartile range (IQR) of OD/cut-off values. The Kruskal–Wallis test and Dunn´s multiple comparison test were used to compare the distribution of binding antibody reactivity against recombinant polypeptides C2V3C3 from different clades. The non-parametric Mann–Whitney U test was used to compare the distribution of antibody binding titers. Spearman’s rank correlation coefficient (r) was used to analyze the association between antibody binding responses and percentage of neutralization in mice sera and the associations between Tfh and Tfr cell counts and binding antibodies levels or neutralizing activity. Comparison between groups was done using the two-tailed non-parametric Mann-Whitney U test. All hypothesis tests were two-tailed, and *p* values <0.05 were considered significant. 

### 2.12. Accession Number(s)

The nucleotide sequence data determined during the course of this work were deposited in GenBank under the following accession numbers: KU310618 to KU310620 and MH045987 to MH045989.

## 3. Results

### 3.1. Generation of Recombinant Vaccinia Viruses Expressing gp120t from Different HIV-1 Clades

**Figure 1:**
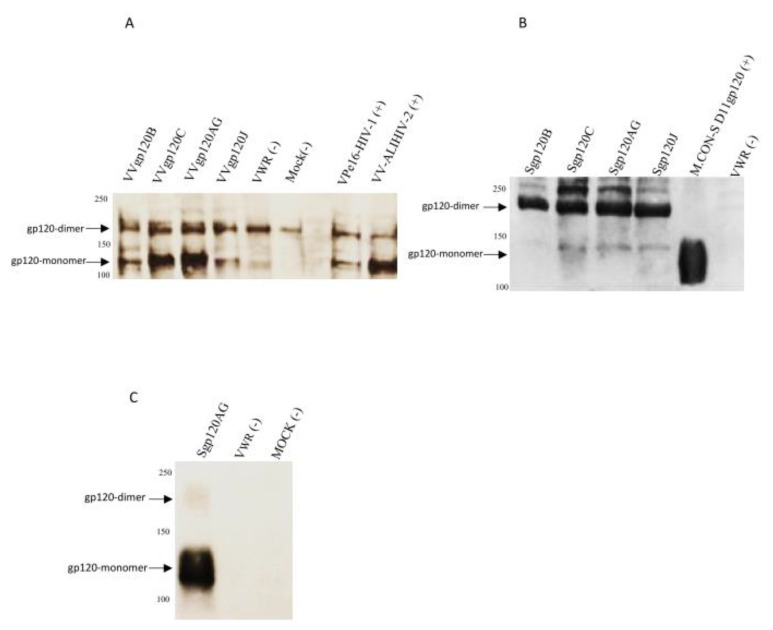
Western blot analysis of envelope gp120t expressed by recombinant Vaccinia viruses. HeLa cells were infected with recombinant Vaccinia viruses expressing envelope gp120t from HIV-1 clades B (VVgp120B), C (VVgp120C), J (VVgp120J), and CRF_AG (VVgp120AG). Cell lysates (**A**) and cell supernatants (**B**) were incubated with sera from HIV-1 infected individuals. Supernatant from cells infected with VVgp120AG was also incubated with bNAb 447-52D (anti-V3) (**C**). Positive controls for these experiments were recombinant Vaccinia viruses expressing the HIV-1 envelope (vPE16), the HIV-2 envelope (rVV/ALI), and a consensus group M envelope gp120 (M.CON.S-D11). Negative controls were uninfected cells (mock) and cells infected with the WR strain of Vaccinia virus (V_WR_). Molecular weight is indicated in KDa on the left.

The new HIV-1 envelope sequences produced in this study belong to clades B, C, G, H, J, and CRF02_AG, as determined by phylogenetic analysis (Figure S1). All HIV-1 isolates used the CCR5 co-receptor as determined phenotypically and/or genotypically from the V3 sequence. Recombinant Vaccinia viruses expressing the truncated gp120 from all clades were obtained with infectious titers in the range of 10^7^–10^9^ PFU/mL. WB analysis with plasma from HIV-1-infected patients demonstrated the presence of monomeric gp120t glycoproteins (~120kDa) in HeLa-CCL2 cells infected with the recombinant viruses (Figure 1). The amount of gp120t recovered from cell supernatants ranged from 63 to 129 µg/mL. No gp160 or gp41 was observed as expected. In the WB analysis of cell extracts and supernatants, there is a strongly reactive band with ~181kDa that corresponds to gp120-dimers. Previous studies have shown that the expression of recombinant gp120 usually produces a substantial amount of disulfide-linked gp120 dimer, in which gp120 inner domain epitopes and the coreceptor binding surface are occluded [55,56]. Like in our WBs, these dimers are highly antigenic and can be present in higher proportion relative to the monomer. The V1/V2 and V3 regions contribute to dimer formation and are occluded in these dimers. The positive control M.CON-SA11gp120(+) does not show dimer bands because it has a deletion of 11 amino acids at the N-terminal region that eliminates dimer formation [56]. Expression of clade H gp120 was weak (not shown) precluding the use of this recombinant Vaccinia virus in subsequent immunization experiments.

### 3.2. New Envelope Glycoproteins Bind to V3-specific and CD4bs-specific Neutralizing Monoclonal Antibodies

**Figure 2:**
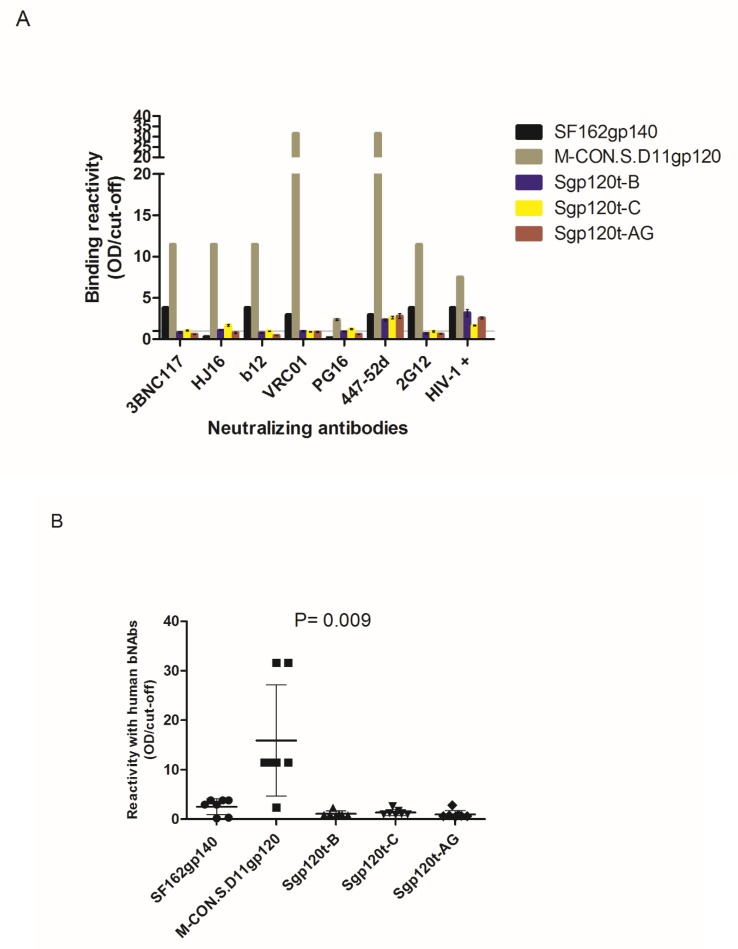
Binding reactivity of human monoclonal antibodies against soluble truncated gp120 glycoproteins and reference glycoproteins. (**A**) Bar graphic showing the antibody reactivity (mean and standard deviation) of the different envelope gp120s against each human monoclonal antibodies and serum from HIV-1 infected individual; (**B**) Dot-plot graphic comparing the mean reactivity and of each envelope glycoprotein against all monoclonal antibodies. Error bars are standard deviation. One-way ANOVA test with Dunn’s Multiple Comparison Test was used to compare the reactivity of the different monoclonal antibodies against the envelope glycoproteins.

The antigenic structure of soluble Sgp120t glycoproteins was assessed by measuring their binding reactivity in ELISA assays against sera from HIV-1-infected patients and human monoclonal antibodies targeting the CD4bs, V2, V3, and N-linked glycans in C2, C3, V4, and C4 regions. Antibodies from HIV-1-infected patients and monoclonal antibody 447-52D bound strongly to all Sgp120t glycoproteins (Figure 2). Binding reactivity of monoclonal antibodies was significantly higher against the M.CON-SD11gp120 reference glycoprotein relative to the B and AG sgp120s (*p* < 0.05) which is likely related to the higher exposure of antibody epitopes due to the deletion of 11 amino acids at the N-terminal region that eliminates dimer formation [56]. There were no significant differences between the binding reactivity of the different Nabs against the three novel gp120s. 447-52D also bound to Sgp120t-AG in Western blot analysis (Figure 1C). These results show that the novel gp120 glycoproteins have a good exposition of the V3 loop.

Amino acid sequences of the new envelope glycoproteins were inspected to try to explain their antigenic profile. The most divergent regions regarding sequence and number of amino acids were V2 (126C-196C, in HXB2), V3 (296C-331C, HXB2), V4 (385C-418C, HXB2), and the C4Dbs beta sheet 23/24 (451G-471G, HXB2), whereas the CD4 binding loop (345S-374H, HXB2), loop D (275V-283T), and gp120/gp41 interface (56S-82Q) were the most conserved regions (Figure S2). Interestingly, gp120t-AG, which showed the highest reactivity in WB and ELISA assays with anti-V3 Nab 447-52D, has the GPGR motif in the V3 crown region instead of the GPGQ motif present in the majority of the other viruses. The data also revealed that gp120t-AG has a high probability of glycan occupancy (Pg = 0.79) at position 332 (in HXB2) due to the presence of the motif NVS [57]. 

### 3.3. Antigenicity of the C2V3C3 Polypeptide

**Figure 3:**
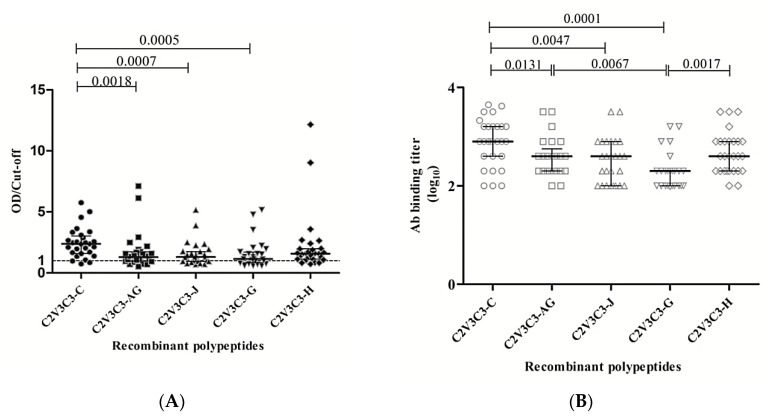
Antibody binding reactivity against recombinant C2V3C3 polypeptides from different subtypes. (**A**) Dot-plot graphic showing the median binding antibody reactivity (OD/Cut-off) and interquartile range of 28 plasma samples from HIV-1 infected individuals against recombinant polypeptides from HIV-1 clades C, G, H, J, and CRF02_AG; (**B**) Dot-plot graphic showing the median binding antibody titers and interquartile range. Kruskal–Wallis and Dunn´s multiple comparison tests were used to compare the distribution of binding antibody reactivity between subtypes. p values < 0.05 were considered statistically significant.

Recombinant polypeptides comprising the C2V3C3 envelope region were obtained for subtypes C, G, H, J, and CRF02_AG with concentrations ranging between 80 and 120 µg/mL. Purified polypeptides reacted positively with plasma form HIV-1-infected patients in Western blot (Figure S3). Antibody binding reactivity and end-point titer were determined in ELISA assays. The majority of plasma samples reacted with the polypeptides in ELISA assays. The lowest reactivity was obtained with subtype J (only 71% of the samples reacted positive) and the highest with subtype C (93%). Median binding titers (log_10_) ranged from 2.151 for subtype G to 2.903 for subtype C (Figure 3). The higher antigenic reactivity of clade C polypeptide is likely related with the predominance of HIV-1 subtype C in Angola where the plasma samples came from [58].

### 3.4. Envelope Glycoproteins Elicit Cross-reactive gp120-specific Binding Antibodies in Immunized Animals

BALB/c mice and New Zealand White rabbits were used to investigate the combined immunogenicity of the new proteins and recombinant Vaccinia viruses. Mice were inoculated in two independent studies, named pilot and main, with several combinations of recombinant Vaccinia viruses expressing gp120t, soluble gp120t (Sgp120t), and recombinant C2V3C3 polypeptides from different clades (Table 1; Table 2), whereas New Zealand white rabbits were immunized only with CRF02_AG-based immunogens (Table 3). Binding antibody responses were analyzed in mice sera drawn at five different time points (T0-T4) using an ELISA assay with Sgp120t or recombinant C2V3C3 polypeptides as capture antigens. For all immunized animals, binding antibody responses were higher than those from control animals. In the pilot study, all animals developed antibodies that reacted with autologous and heterologous Sgp120t glycoproteins soon after the priming with no significant differences between subtypes. In contrast, the majority of immunized mice developed binding antibodies against C2V3C3 polypeptides only after the third immunization and at low levels except in mice boosted with clade C polypeptide eluted in urea and Sgp120t-C (Group 4) (Figure S4). 

**Figure 4:**
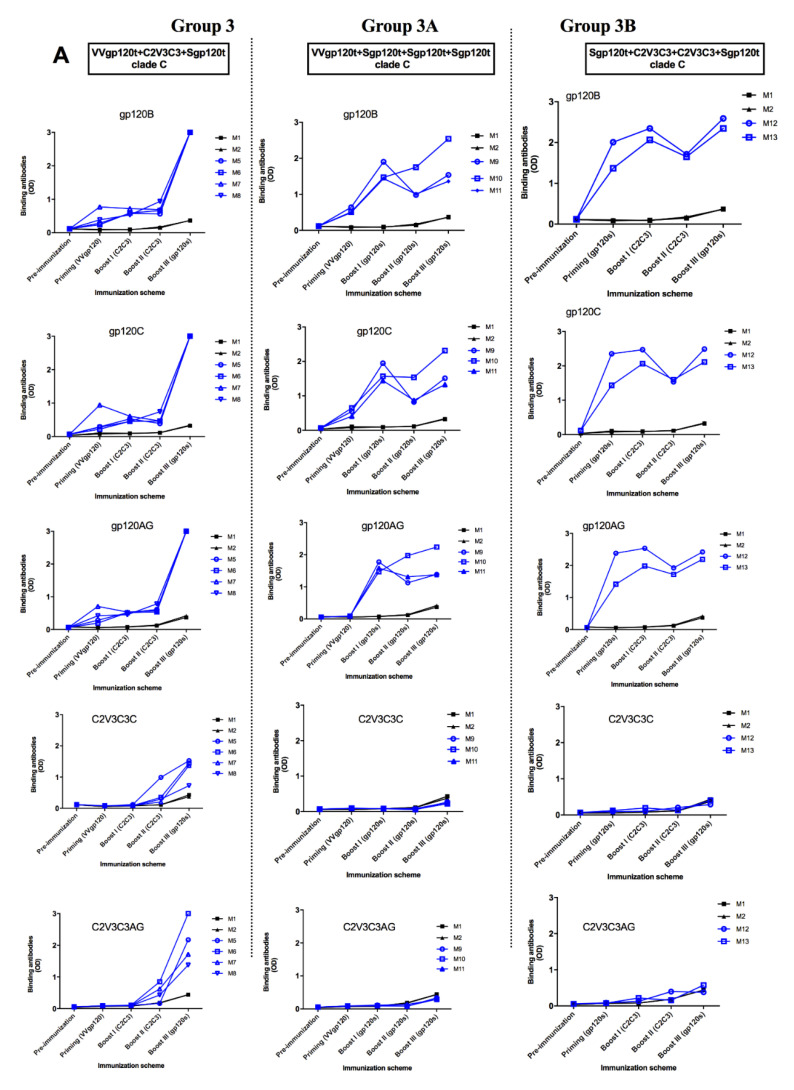

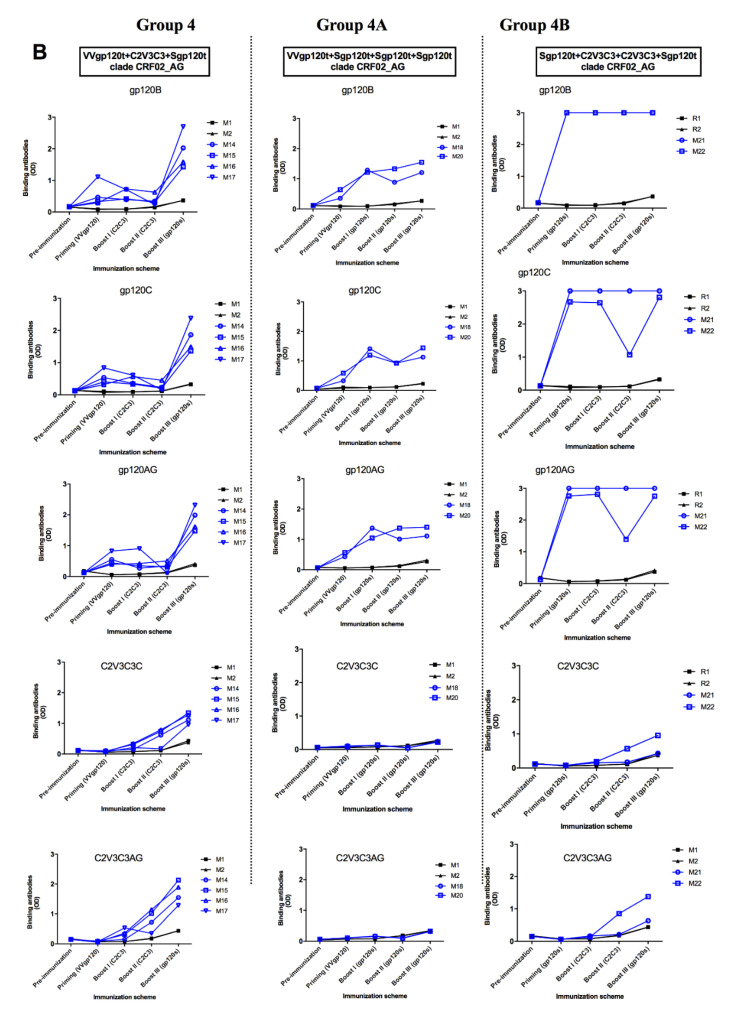

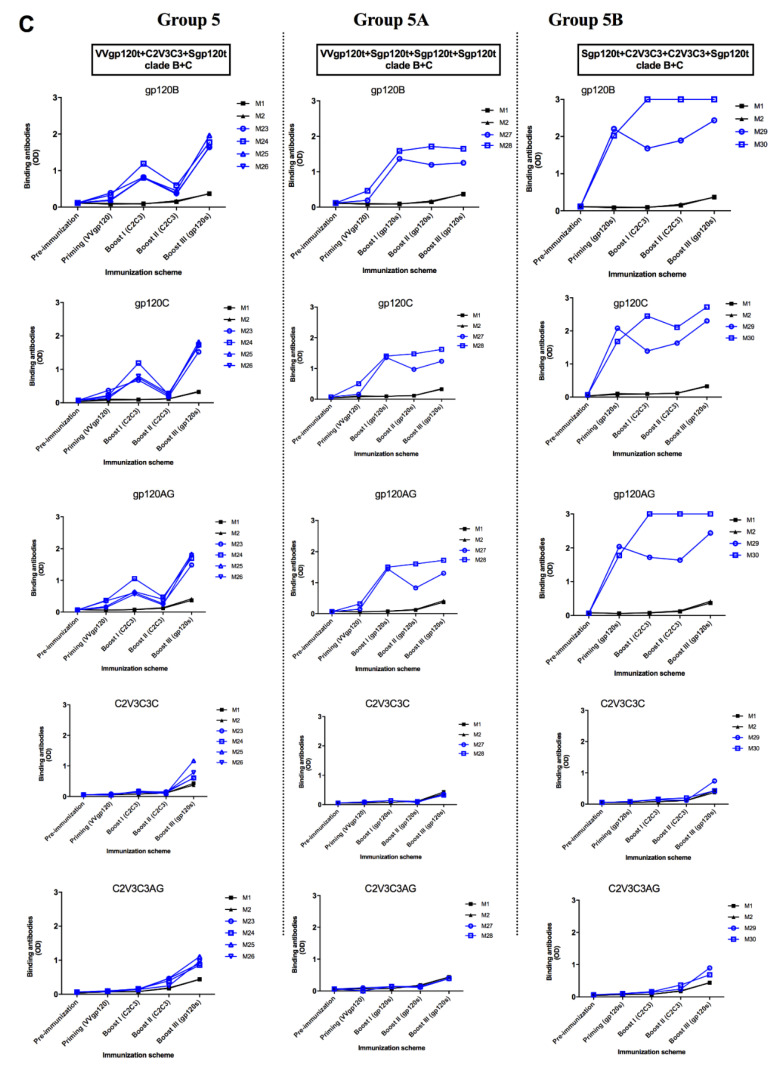
Evolution of the antibody binding responses against soluble gp120ts (clades B, C, CRF02_AG) and C2V3C3 polypeptides (clades C and CRF02_AG) in BALB/c mice immunized with different combinations of HIV-1 immunogens (main study): (**A**) Mice immunized with clade C immunogens; (**B**) Mice immunized with clade CRF02_AG immunogens; (**C**) Mice immunized with clades B (gp120) and C (C2V3C3) immunogens. Groups 3, 4, and 5—mice primed with VV expressing gp120t and boosted with C2V3C3 polypeptides and Sgp120t; Groups 3A, 4A, and 5A—mice primed with VV expressing gp120t and boosted with Sgp120t; Groups 3B, 4B, and 5B—mice primed with Sgp120t and boosted with C2V3C3 polypeptides and Sgp120t. For all mice, the schedule of immunization included one priming and three boosts at days 15, 30, and 45. Fifteen days after each immunization, sera were collected and assayed for the presence of binding antibodies against HIV-1 immunogens. Blue lines represent immunized mice from the respective group; black lines represent mice from control group (G1-M1-M2).

Similarly, in the main mice vaccination study (immunization scheme shown in Table 2), all animals generated IgG antibodies that bound to both autologous and heterologous Sgp120t glycoproteins soon after the priming (Figure 4). Three boosts were necessary to achieve the highest levels of reactivity in both studies. Of note, mice primed with Sgp120t (Groups 3B, 4B, and 5B) generated IgG antibodies with higher binding reactivity relative to mice primed with recombinant Vaccinia viruses. Mice immunized with C2V3C3 polypeptides developed a moderate binding IgG response against C2V3C3 polypeptides from different clades. Similar to the pilot study, mice immunized with clade C immunogens developed the strongest binding response after the third immunization, indicating that C2V3C3 antibodies arise slowly. Interestingly, mice immunized with recombinant Vaccinia viruses and boosted three times with homologous gp120t (Groups 3A, 4A, and 5A) did not produce antibodies against C2V3C3 indicating that epitopes present in the C2V3C3 polypeptides were not presented in gp120 immunogens (Figure 4). Overall, there were no significant differences between immunogen responses, i.e., groups that were immunized with an immunogen from a specific subtype did not produce more binding antibodies to the autologous proteins compared with heterologous proteins. 

**Figure 5:**
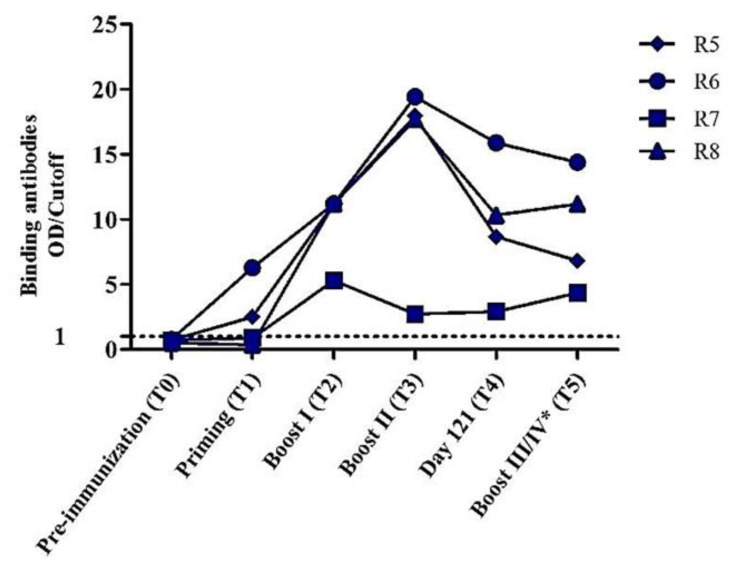
Evolution of the binding IgG reactivity in sera of immunized rabbits against heterologous protein HIV-1 group M consensus gp120 envelope (M. CON-S D11). Animals R5–8 (Group 3) were primed with VVgp120t-AG and boosted with Sgp120t-AG. Priming was administered at day 1 and boosts I, II, and III at days 35, 63, and 121, respectively. *Animals R6 and R8 received an extra boost at day 150 (boost IV). All animals were sacrificed at day 154 (T5). Cut-off value was calculated as the mean OD of rabbits immunized with VV_WR_ immunogens (control Group 2) plus four times the standard deviation. OD/cut-off ≥1 was considered positive and is defined by the dashed line.

**Figure 6:**
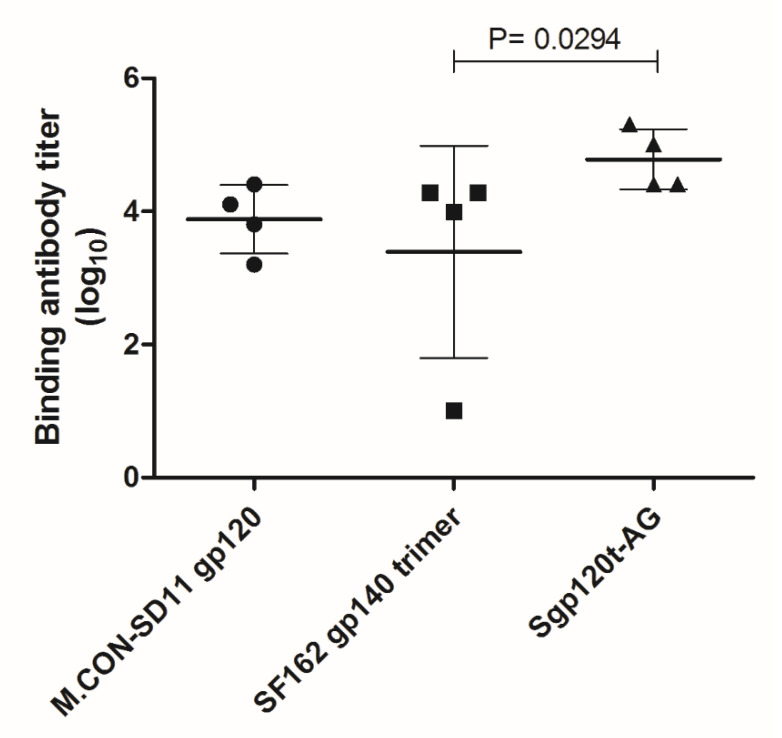
IgG binding antibody titers in vaccinated rabbits against homologous and heterologous envelope glycoproteins at day 154. Median antibody titers were compared using the Mann–Whitney U test. The lines represent mean and standard deviation values.

Regarding the study in rabbits, binding antibody reactivity (G3) was much higher in vaccinated animals relative to animals in the control groups (G1 and G2) (Figure 5). All immunized rabbits from Group 3 (R5–R8) developed specific antibodies against the heterologous gp120 M.CON.SD11 after the first boost. Except for R7, binding IgG responses increased up to day 99, followed by a slight decline at day 121. At the final day (154), all animals had high IgG binding antibody titers against the autologous gp120 and heterologous Env glycoproteins (Figure 6). Rabbit antibodies also reacted strongly with the monomeric and dimeric forms of homologous Sgp120t-AG and multiple heterologous gp120s in WB (Figure S5). Monomeric and dimeric forms were also observed with lectin-affinity purified gp120t-AG (Figure S5). Overall, these results demonstrate that the novel CRF02_AG immunogens elicit the production of antibodies in rabbits that bind strongly to different HIV-1 Env trimers, dimers, and monomers. 

### 3.5. Production of Heterologous Tier 2 HIV-1 Neutralizing Antibodies in Immunized Animals

In the mice experiments, neutralizing antibodies against tier 2 viruses from different clades were produced in at least one animal per group (Table 4; Table 5). In the pilot study, animals immunized with clade C, were able to neutralize >50% (at 1:40 sera dilution) two heterologous tier 2 pseudoviruses from clades A and C and one primary isolate from clade J. One animal immunized with clade J neutralized >50% one heterologous tier 2 pseudovirus from clade C. Notably, one animal immunized with CRF01_AG-derived immunogens (M13, Group 5) elicited neutralizing antibodies against four tier 2 pseudoviruses and two primary isolates from different clades (Table 4). Overall, the percentage of neutralized tier 2 viruses per group was 23% (3/13) for Group 3 (immunized with clade C), 46% (6/13) for Group 5 (immunized with clade AG), and 8% (1/13) for Group 6 (immunized with clade J).

In the mice main study, at least one mouse per main group (immunized with clades C, AG, and B proteins) neutralized at least one tier 2 virus at more than 50%, including viruses from different clades (Table 5). However, there were significant differences in breadth and potency of neutralization depending on the immunogens. Mice primed with VVgp120t-AG and boosted with the homologous Sgp120t-AG (Group 4A, main study) produced the most potent and broad neutralizing responses (>49%), which included two HIV-1 primary isolates, one heterologous (isolate 93HDC253, clade J), one autologous (isolate 01PTCJN, clade AG), two heterologous tier 2 pseudoviruses (clades B and CRF07_BC), and one tier 1 isolate (SG3.1) (Table 5). Mice primed with Sgp120t-B and boosted with C2V3C3-C polypeptide (Groups 5, 5A, and 5B, main study) had low neutralizing responses with only one group (M23-M26, Group 5) neutralizing one virus at more than 50% (Table 5). Sera from animals immunized with clade C immunogens (Group 3, both studies), neutralized one heterologous tier 2 pseudovirus (clade B) and the clade AG primary isolate (Table 4; Table 5). Overall, the weakest neutralizing responses were observed in animals primed with Sgp120t (Groups 3B, 4B, and 5B, main study) instead of recombinant VV (Table 5). These groups achieved the higher IgG binding response against all soluble gp120 glycoproteins indicating that the majority of antibodies generated were non-neutralizing (Figure 4). Overall, the percentage of tier 2 viruses neutralized per group was 21% (3/14) for mice immunized with clade C-derived immunogens (Groups 3, 3A, 3B, main study), 43% (6/14) for mice immunized with clade AG-derived immunogens (Groups 4, 4A, and 4B, main study), and 7% for mice immunized with clade B+C-derived immunogens (5, 5A, 5B, main study). Tier 2 viruses BJOX2000 (clade CRF07), PCE1176 (clade C), and 246F3 (clade AC) were not neutralized by any sera in both studies. Both experiments in mice (pilot and main study) generated coherent and reproducible results in that the animals immunized with clade AG-derived immunogens produced the better neutralizing responses.

Regarding neutralization assays in rabbits, sera from 2 of 4 animals primed with recombinant VVgp120t-AG isolate and boosted with the homologous Sgp120t-AG (R7 and R8) neutralized ≥5 tier 2 viruses (>30% neutralization) (Table 6). This neutralization was HIV-1-specific as determined by the lack of neutralization of HIV-2 primary isolates and VSV-pseudotyped viruses. Notably, one animal (R8) was able to neutralize at >50% almost the entire tier 2 virus panel (13/16, 81.3%) at titers ranging from 1:20 (11 isolates) to 1:80 (1 isolate). In addition, four of the pseudoviruses from the panel and three primary isolates were neutralized at >80% by this particular animal (Table 6). 

## 4. Discussion

We show here that mice and rabbits primed with VV expressing gp120t-AG and boosted with the homologous soluble gp120t produced antibodies that bind to homologous and heterologous envelope glycoproteins and, in some cases, neutralize different HIV-1 tier 2 clades (including primary isolates and pseudoviruses). Mice developed neutralizing responses >49% against 4/14 (29%) tier 2 HIV-1 isolates of clades B, CRF07_BC, J, and CRF02_AG (homologous virus). Notably, one rabbit developed potent neutralizing responses against almost all tier 2 HIV-1 viruses tested (13/16, 81.3%), although at low titers (1:20–1:80). Neutralized isolates were highly diverse belonging to clades A, B, C, G, J, U, AC, CRF01_AE, CRF02_AG, and CRF07_BC. A similar strategy using a replication-competent Vaccinia virus expressing the full Env (gp160) plus two gp120 subunit boosts of clade B virus was able to elicit cross-reactive neutralizing activities against >50% of a global panel of tier 2 HIV-1 isolates in rabbits [29]. Like in our study, the neutralizing response was observed only in some animals and at low titers (1:20–1:100). The observation that only animals primed with Vaccinia virus developed bNAbs emphasizes the importance of choosing the right prime-boost immunization regimen and suggests a possible role for replication-competent virus in HIV-1 vaccine design [29,30,59]. Consistent with this, a recent study in non-human primates (NHPs) has shown that priming with replication-competent attenuated Vaccinia virus strain NYVAC expressing clade C gp140 elicited significantly higher titers of binding and neutralizing antibodies relative to priming with replication-deficient NYVAC expressing the same clade C gp140 [38,60].

Immunogens used in our study were mostly derived from R5 isolates from HIV-1 infected patients from Angola. We hypothesized that envelope glycoproteins from viruses from an old epidemic, such as the HIV epidemic in Angola, would be better at eliciting bNAbs against heterologous viruses as they comprise key epitopes and conformational determinants that should be conserved in the contemporaneous strains due to functional constraints [41,61,62]. Our results seem to confirm this hypothesis and provide the first demonstration that a vaccine based on envelope immunogens from HIV-1 clade CRF02_AG can generate neutralizing antibodies against highly divergent tier 2 HIV-1 isolates and pseudoviruses. 

In previous studies in mice, we showed that a strong and broad NAb response against HIV-2 isolates can be elicited in mice primed with soluble gp125t expressed in vaccinia virus and boosted with a C2V3C3 polypeptide produced in *E. coli* (rpC2–C3) [30]. We therefore hypothesized that a similar neutralizing response could be elicited for HIV-1 using the same immunogens and immunization strategy. Our current results show that this is not the case for HIV-1, indicating that C2V3C3 polypeptides from HIV-1 are unable to effectively present neutralizing epitopes and boost the neutralizing responses and suggesting that there are major differences in the antigenic structure of this variable region between HIV-1 and HIV-2 [63]. 

Potent binding antibody responses (i.e., high level and able to bind to homologous and cross-clade heterologous envelope glycoproteins) were generated in all mice and rabbits, with log_10_ titers in rabbits ranging from 3.393 to 4.785, which are similar to those obtained in recent human vaccination trials using two different vaccine concepts [27,64]. Higher IgG binding responses were found against gp120s from different clades (B, C, and CRF02_AG) in animals that were primed with soluble gp120 instead of recombinant Vaccinia virus. However, these animals had the weakest neutralizing responses which corroborates previous findings and further demonstrates that immunization with monomeric/dimeric gp120 or other monomeric/dimeric Env proteins alone is generally not enough to induce clinically relevant neutralizing antibodies against HIV-1 [3,23,30,65,66,67,68,69,70].

To initially address the question why CRF02_AG envelope glycoproteins elicited more potent neutralizing responses relative to envelope glycoproteins from other clades, we analyzed the amino acid sequence of all envelopes. The observation that gp120-AG had a GPGR motif in the V3 crown was interesting, since the GPGQ motif is far more common in non-B clades [17,71,72,73,74]. Related to this, 447-42D was the only monoclonal antibody that bound to gp120t-AG both in ELISA and WB. This antibody neutralizes preferentially isolates with GPGR motif in the V3 crown and has limited capacity against isolates with the GPGQ motif [16,17,71]. This may indicate that gp120t-AG-based immunogens elicit the production of neutralizing antibodies targeting the V3 crown. A second epitope in V3 may also have been involved in neutralization. The glycan supersite centered on N332 in the base of the V3 loop is a vulnerable site for neutralization by some bNAbs (e.g., PGT121 and PGT128) [57,75,76]. The second amino acid (X) in the canonical N-linked glycosylation motif NXS/T indicates whether or not a glycan will be attached to it [57,77,78]. gp120t-AG has a high probability of N332 glycan occupancy (Pg = 0.79, motif NVS) and X2278, the clade B isolate neutralized by all gp120t-AG-immunized mice and rabbit 8, also has a high probability of N332 glycan occupancy (Pg = 0.86, motif NIS). This may indicate that gp120t-AG immunogens elicit bNAbs targeting the glycan supersite N332 in both animals. Experimental studies will be necessary to identify the epitope specificities of NAbs elicited by the new gp120-AG immunogens. 

## 5. Conclusions

We have shown that a prime-boost vaccination strategy based on recombinant Vaccinia viruses expressing a novel gp120 derived from a CRF02_AG isolate is able to elicit in mice and rabbits the production of antibodies that bind to homologous and heterologous envelope glycoproteins. In addition, antibodies that neutralize diverse tier 2 HIV-1 isolates, including primary isolates, were elicited in a subset of the animals. These results suggest that the novel CRF02_AG-based immunogens and prime-boost immunization strategy may be able to elicit the type of immune response required for a preventive HIV-1 vaccine.

## Figures and Tables

**Table 1:** BALB/c mice immunization scheme in the pilot study.

Groups	Mice ID	HIV-1 Env Clade	Priming	Boost I	Boost II	Boost III
			Day 1	Day 15	Day 30	Day 45
**1**	M1-M2	-	-	-	-	-
**2**	M3-M5	-	VV_WR_	-	-	-
**3**	M6-M8	C	VVgp120t-C	C2V3C3-C^a^	C2V3C3-C^a^	Sgp120t-C
**4**	M9-M11	C	VVgp120t-C	C2V3C3-C^b^	C2V3C3-C^b^	Sgp120t-C
**5**	M12-M16	CRF02_AG	VVgp120t-AG	C2V3C3-AG	C2V3C3-AG	Sgp120t-AG
**6**	M17-M20	J	VVgp120t-J	C2V3C3-J	C2V3C3-J	Sgp120t-J

A total of 20 female BALB/mice divided into six groups were first immunized intraperitonealy (IP) with 2 × 10^7^ PFU of recombinant Vaccinia viruses expressing gp120t from clades C, CRF02_AG, and J and boosted with 10 µg of C2V3C3 and 7–12.9 µg of the homologous Sgp120t. Each animal was immunized at days 1, 15, 30, and 45. Mice sera were collected 15 days after each immunization. All mice were sacrificed at day 60. Groups 1 and 2 were control groups; VV_WR_—wild-type Vaccinia.

**Table 2:** BALB/c mice immunization scheme in the main study.

Groups	Mice ID	HIV-1 Env Clade	Priming	Boost I	Boost II	Boost III
			Day 1	Day 15	Day 30	Day 45
**1**	M1-M2	-	-	-	-	-
**2**	M3-M4	-	VV_WR_	S_WR_	S_WR_	S_WR_
**3**	M5-M8	C	VVgp120t-C	C2V3C3-C	C2V3C3-C	Sgp120t-C
**3A**	M9-M11	C	VVgp120t-C	Sgp120C	Sgp120C	Sgp120t-C
**3B**	M12-M13	C	Sgp120t-C	C2V3C3-C	C2V3C3-C	Sgp120t-C
**4**	M14-M17	CRF02_AG	VVgp120t-AG	C2V3C3-AG	C2V3C3-AG	Sgp120t-AG
**4A**	M18-M20	CRF02_AG	VVgp120t-AG	Sgp120t-AG	Sgp120t-AG	Sgp120t-AG
**4B**	M21-M22	CRF02_AG	Sgp120t-AG	C2V3C3-AG	C2V3C3-AG	Sgp120t-AG
**5**	M23-M26	B	VVgp120t-B	C2V3C3-C	C2V3C3-C	Sgp120t-B
**5A**	M27-M28	B	VVgp120t-B	Sgp120t-B	Sgp120t-B	Sgp120t-B
**5B**	M29-M30	B	Sgp120t-B	C2V3C3-C	C2V3C3-C	Sgp120t-B

A total of 30 mice divided into 11 groups were immunized intraperitonealy with different combinations of immunogens: 2 × 10^7^ PFU of recombinant Vaccinia virus expressing gp120t from HIV-1 clades B, C, and CRF02_AG in 100 µL of PBS; 10 µg of recombinant polypeptides C2V3C3-C and C2V3C3-AG and 7–12.9 µg of homologous Sgp120t. Each animal was immunized at days 1, 15, 30, and 45. Mice sera were collected fifteen days after each immunization. All mice were sacrificed at day 60. Groups 1 and 2 were control groups; VVWR—wild-type Vaccinia virus strain Western Reserve; SWR—supernatant of HeLa cells infected with VVW.2.9. New Zealand White Rabbits Immunization

**Table 3:** Rabbit immunization schedule.

Groups	ID	Blood Collection	Priming	Blood Collection	Boost I	Blood Collection	Boost II	Blood Collection	Blood Collection	Boost III	Boost IV	Blood Collection
		Day 1 (T0)	Day 1	Day 35 (T1)	Day 35	Day 63 (T2)	Day 63	Day 99 (T3)	Day 121 (T4)	Day 121	Day 150	Day 154 (T5)
1	R1	●	-	●	-	●	-	●	●	-	-	●
	R2	●	PBS	●	PBS	●	PBS	●	●	PBS	-	●
2	R3	●	VV_WR_	●	S_WR_	●	S_WR_	●	●	S_WR_	-	●
	R4	●	VV_WR_	●	S_WR_	●	S_WR_	●	●	S_WR_	-	●
3	R5	●	VVgp120t-AG	●	Sgp120t-AG	●	Sgp120t-AG	●	●	Sgp120t-AG	-	●
	R6	●	VVgp120t-AG	●	Sgp120t-AG	●	Sgp120t-AG	●	●	Sgp120t-AG	Sgp120t-AG	●
	R7	●	VVgp120t-AG	●	Sgp120t-AG	●	Sgp120t-AG	●	●	Sgp120t-AG	-	●
	R8	●	VVgp120t-AG	●	Sgp120t-AG	●	Sgp120t-AG	●	●	Sgp120t-AG	Sgp120t-AG	●

VV_WR_—wild-type Vaccinia virus strain Western Reserve; S_WR_—supernatant of cells infected with VV_WR_; VVgp120t-AG—Vaccinia virus expressing gp120t from HIV-1 clade CRF02_AG; Sgp120t-AG—soluble gp120t expressed from VVgp120t-AG; PBS—phosphate-buffered saline; ID—animal identification; R—rabbit.

**Table 4:** Neutralizing activity in mice immunized in the pilot mice study.

Immunization Scheme	Mice ID	Group	Immunogen Clade	Tier 1 Isolate	Tier 2 Pseudovirus Panel											Primary Isolates	
				NL4.3	398F1	X2278	TRO11	25710	CE0217	CE1176	X1632	CNE55	CH119	BJOX2000	246F3	01PTCJN	93HDC250
				B	A	B	B	C	C	C	G	CRF07_BC	CRF07_BC	CRF07_BC	AC	CRF02_AG	J
Mock control	M1-M2	1	_	<30	<30	<30	<30	<30	<30	<30	<30	<30	<30	<30	<30	<30	<30
Vaccinia control (VV_WR_+S_WR_)	M3-M5	2	_	<30	<30	<30	<30	<30	<30	<30	<30	<30	<30	<30	<30	<30	<30
VVgp120t+C2V3C3+C2V3C3+Sgp120t	M6	3	C	58	69	<30	<30	59	35	<30	<30	<30	<30	<30	<30	<30	<30
	M7			ND	61	<30	<30	ND	47	<30	<30	41	<30	<30	<30	<30	<30
	M8			<30	<30	<30	<30	<30	<30	<30	<30	<30	<30	<30	<30	<30	50
	M9	4	C	ND	47	ND	31	ND	ND	<30	ND	ND	ND	ND	ND	ND	ND
	M10			49	<30	<30	<30	<30	<30	<30	<30	<30	<30	<30	<30	<30	<30
	M11			ND	<30	<30	<30	ND	<30	<30	<30	<30	<30	<30	<30	<30	<30
	M12	5	CRF02_AG	<30	37	<30	<30	<30	<30	<30	<30	<30	<30	<30	<30	<30	<30
	M13			ND	76	ND	<30	ND	56	<30	50	57	ND	47	<30	49	55
	M15			ND	<30	<30	<30	ND	<30	<30	<30	<30	<30	<30	<30	<30	<30
	M16			<30	<30	<30	<30	<30	<30	<30	<30	<30	<30	<30	<30	<30	<30
	M17	6	J	<30	<30	<30	<30	<30	<30	<30	<30	ND	ND	<30	<30	ND	<30
	M18			<30	<30	<30	<30	<30	<30	<30	<30	<30	<30	<30	<30	<30	<30
	M19			73	<30	<30	<30	54	<30	<30	<30	<30	<30	<30	<30	<30	<30
	M20			ND	<30	<30	<30	ND	<30	<30	<30	<30	<30	<30	<30	<30	<30
Control of neutralization*				95	78	48	69	92	63	91	90	67	75	78	75	51	87

Percent neutralization was determined in TZM-bl cells measuring the reduction in number of RLU relative to wells with the corresponding pre-immune sera. Beige highlighting indicates less than 30% of neutralization; salmon highlighting indicates ≥ 30–49% neutralization; red highlighting indicates ≥ 50% of neutralization. ND—not done due to lack of serum. Mice sera were used at 1:40 final dilution. *Sera from an HIV-1-infected individuals with potent neutralizing activity at 1:20 final dilution. S_WR_—supernatant from HeLa cells infected with Vaccinia virus strain WR.

**Table 5:** Neutralizing activity in mice immunized in the main mice study.

Immunization Scheme	Groups	Immunogen Clade	Tier 1 Isolate	Tier 2 Pseudovirus Panel											Primary Isolates			VSV
			SG3.1	398F1	X2278	TRO11	25710	CE0217	CE1176	X1632	CNE55	CH119	BJOX2000	246F3	01PTCJN	93HDC250	93HDC253	
			B	A	B	B	C	C	C	G	CRF07_BC	CRF07_BC	CRF07_BC	AC	CRF02_AG	J	J	
Mock control	1	_	<30	<30	<30	<30	<30	<30	<30	<30	<30	<30	<30	<30	<30	<30	<30	<30
Vaccinia control (VV_WR_+S_WR_)	2	_	<30	<30	<30	<30	<30	<30	<30	<30	<30	<30	<30	<30	<30	<30	<30	<30
VVgp120t+C2V3C3+C2V3C3+Sgp120t	3	C	<30	<30	40	52	<30	<30	<30	<30	<30	34	<30	<30	54	<30	<30	<30
VVgp120t+Sgp120t+Sgp120t+gp120t	3A		<30	<30	42	<30	<30	<30	<30	<30	<30	ND	<30	<30	46	ND	ND	<30
Sgp120t+C2V3C3+C2V3C3+Sgp120t	3B		<30	<30	55	<30	<30	<30	<30	<30	<30	<30	<30	<30	<30	<30	<30	<30
VVgp120t+C2V3C3+Sgp120t	4	CRF02_AG	<30	<30	53	<30	<30	<30	<30	<30	<30	<30	<30	<30	<30	<30	<30	<30
VVgp120t+Sgp120t+Sgp120t+Sgp120t	4A		56	<30	52	44	<30	<30	<30	<30	36	49	<30	<30	70	<30	50	<30
Sgp120t+C2V3C3+C2V3C3+Sgp120t	4B		44	38	51	<30	<30	<30	<30	<30	<30	ND	<30	<30	ND	ND	ND	<30
VVgp120t+C2V3C3+Sgp120t	5	B+C	<30	<30	<30	<30	<30	<30	<30	<30	<30	48	<30	<30	58	<30	<30	<30
VVgp120t+Sgp120t+Sgp120t+gp120t	5A		<30	43	<30	ND	<30	<30	<30	<30	35	ND	<30	<30	44	ND	ND	<30
Sgp120t+C2V3C3+C2V3C3+Sgp120t	5B		<30	<30	<30	<30	<30	<30	<30	<30	<30	<30	<30	<30	<30	<30	<30	<30
Control of neutralization^*^			99	67	93	69	92	94	91	80	95	57	78	95	51	38	75	<30

Percent neutralization was determined in TZM-bl cells measuring the reduction in number of RLU relative to wells with the corresponding preimmune sera. Beige highlighting indicates less than 30% of neutralization; salmon highlighting indicates ≥ 30%–49% neutralization; red highlighting indicates ≥ 50% of neutralization; ND—not done due to lack of serum. Mice sera were used at 1:40 final dilution. *Sera from an HIV-1-infected individuals with potent neutralizing activity at 1:20 final dilution. VSV—pseudotyped viruses were used as neutralization specificity control. S_WR_—supernatant from HeLa cells infected with Vaccinia virus strain WR.

**Table 6:** Neutralizing activity of rabbit sera immunized with CRF01_AG-derived immunogens^1^

Immunization Scheme and Controls	Animal ID	HIV-1 Pseudoviruses														Primary Isolates				Specificity Controls		
		Tier 1		Tier 2												HIV-1				HIV-2 Isolates		VSV
		NL 4.3	SG 3.1	PCE 0217	PCNE8	PCH 119	TRO 11	CNE 55	CE 1176	246 F3	BJOX 2000	X 1632	X 2278	398 F1	25 710	01PT CJN	93HD C252	93HD C253	93HD C249	HCC 19.03	AUC	
		B	B	C	01 _AE	07_BC	B	07 _BC	C	AC	07 _BC	G	B	A	C	02 _AG	U	J	U	A	A	
Mock	R1-R2	<30	<30	<30	<30	<30	<30	<30	<30	<30	<30	<30	<30	<30	<30	<30	<30	<30	<30	<30	<30	<30
Vaccinia (VV_WR_+S_WR_)	R3-R4	<30	<30	<30	<30	<30	<30	<30	<30	<30	<30	<30	<30	<30	<30	<30	<30	<30	<30	<30	<30	<30
VV gp120t- AG+Sgp120t-AG	R5-R6	<30	<30	<30	<30	<30	<30	<30	<30	<30	<30	<30	<30	<30	<30	<30	<30	<30	<30	<30	<30	<30
	R7	<30	36	<30	<30	<30	<30	<30	<30	39	<30	45	<30	<30	<30	32	33	<30	<30	<30	<30	<30
	R8	58 (20)	63 (20)	30	90 (20)	36	32	58 (20)	54 (20)	80 (20)	61 (20)	77 (20)	79 (20)	66 (20)	86 (80)	71 (40)	91 (40)	65 (20)	75 (20)	<30	<30	<30
Neutralization controls	bNAbs	94	95	92	70	85	82	62	<30	66	<30	<30	93	79	<30	90	85	84	<30	<30	<30	<30
	HIV-1*	89	82	96	100	98	97	95	95	86	79	81	92	99	99	97	91	92	96	<30	<30	<30

^1^ Percent neutralization was determined for all samples at 1:20 sera dilution in TZM-bl cells measuring the reduction in number of RLU relative to wells with the corresponding pre-immune sera. Beige highlighting indicates less than 30% of neutralization; salmon highlighting indicates ≥ 30%–49% neutralization; red highlighting indicates ≥ 50% of neutralization. For animal R8 neutralization titers were determined for all viruses that were neutralized > 50% at 1:20 dilution, and the results are given between parentheses. bNAbs—Human monoclonal antibodies PG16, HJ16, VRC01 at a final concentration between 0.02–1 µg/mL. HIV-1+—Sera from an HIV-1-infected individual with potent neutralizing activity at 1:20 final dilution. HIV-2 primary isolates and VSV-pseudotyped viruses were used as neutralization specificity control. SWR—supernatant from HeLa cells infected with Vaccinia virus strain WR.

## Supplementary Materials

The following are available online at https://www.mdpi.com/2076-393X/8/2/171/s1. Figure S1: Phylogenetic analysis of HIV-1 env genes used in this study; Figure S2: Amino acid sequence alignment of envelope sequences included in the present study and location of neutralizing epitopes; Figure S3: Western blot analysis of the antigenic reactivity of C2V3C3 polypeptides; Figure S4: Evolution of the binding antibody responses against Sgp120t glycoproteins and C2V3C3 polypeptides in BALB/c mice in the pilot study. Figure S5: Western blot analysis of the reactivity of rabbits’ serum with Sgp120t glycoproteins from different clades; Table S1: Characterization of the nine HIV-1 primary isolates included in the study; Table S2: Primers used for polymerase chain reaction amplification of HIV-1 Env, C2V3C3, and truncated gp120; Table S3: Primers used for sequencing of HIV-1 env gene segments; Table S4: Characteristics of the global panel of twelve tier 2 HIV-1 Env-pseudoviruses used in neutralization assays; Table S5: Baseline data of the neutralization assays performed in mice (main study) (A) and rabbits (B).
