# Supplementary material for: A Prime-Boost Immunization Strategy with Vaccinia Virus Expressing Novel gp120 Envelope Glycoprotein from a CRF02_AG Isolate Elicits Cross-Clade Tier 2 HIV-1 Neutralizing Antibodies
Source: Vaccines (Basel). 2020 Apr 7;8(2):171. doi: 10.3390/vaccines8020171 (PMC7349027; doi:10.3390/vaccines8020171)
Supplement: Supplementary file 1 [file vaccines-08-00171-s001.pdf]

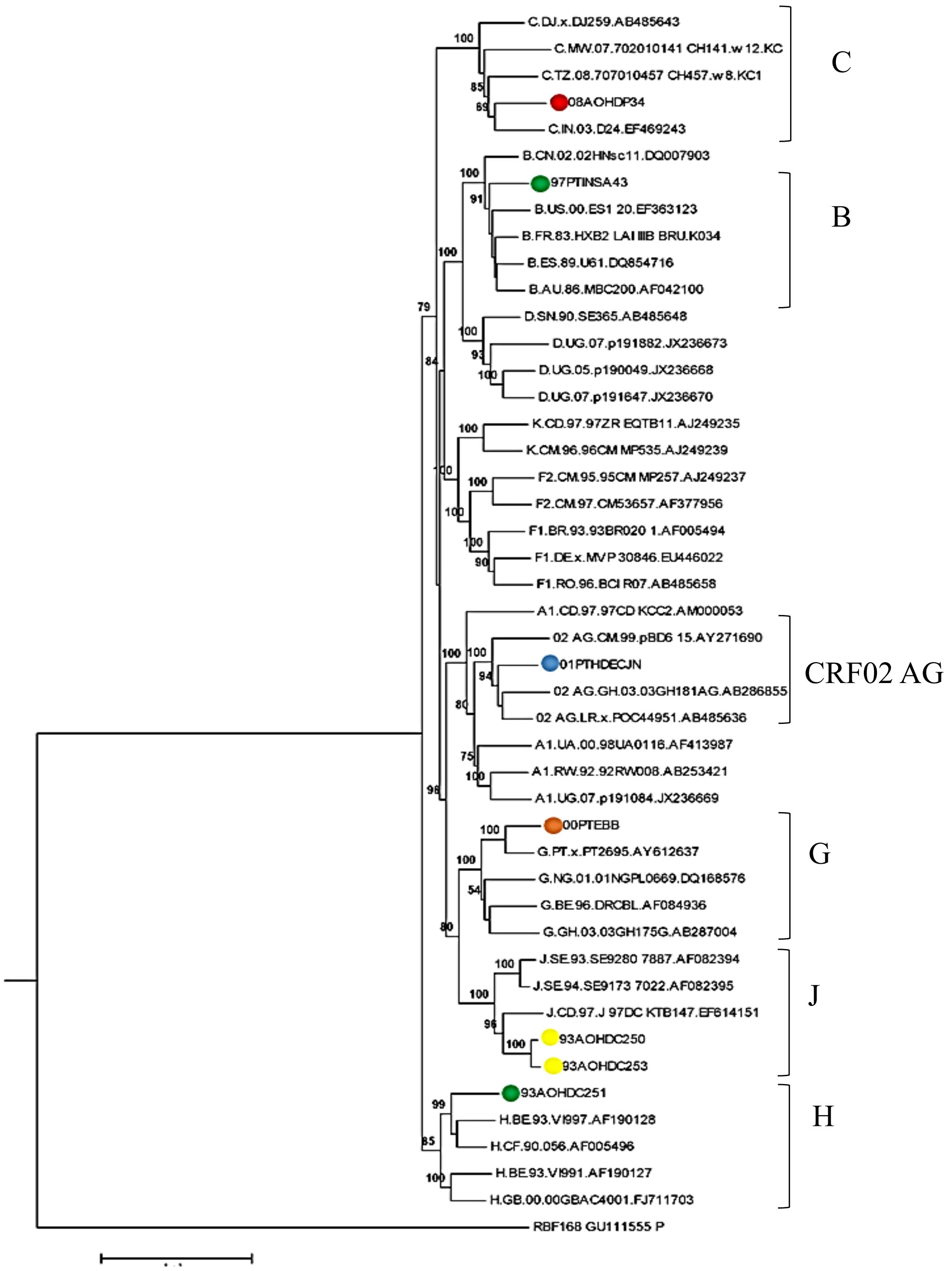

Figure S1 - Phylogenetic analysis of HIV-1 *env* genes used in this study. The maximum likelihood phylogenetic trees were constructed with alignments of reference sequences representative of all HIV-1 group M subtypes and the *env* gene sequences produced in this study (represented with colored dots). Sequence RBF168 GU111555 belonging to HIV-1 group P was used as outgroup. Only bootstrap values above 70% are shown.

```
                                                               gp120/gp41 interface (PGT151)

                      *        20         *        40         *        60         *        80         *       100         *
HXB2       : MRVK---EKYQHLWRWGWRWGTMLLGMLMICSATEKLWVTVYYGVPVWKEATTTLFCASDAKAYDTEVHNVWATHACVPTDPNPQEVVLVNVTENFNMWKNDMVEQMHEDIISLW : 112
JR FL      : ....GIRKN....----..G..L...IIV....V.......................................................G....K......N.....Q....... : 111
93A0HDC250 : .K.METQTSWKS.----....L.IF..F...K...Q...............K..........S.S..G..I...............I..E.......I.E.............V. : 111
08AO34HDP  : ...MGIMRNC.QW----.I..ILGFW......VVGN...............K............EK....................M..G.........E....D.......... : 111
01PTHDECJN : .K.RGIQKNCPL.----.G...IIFWIMI..KT-.D............RD.E..................................IR.E.......I...N......G...... : 110
97PTINSA43 : .K..GIWKNC...----......FIWL...G..A.PR............DT...................................I..E...........N.A........... : 111

                                       V1                    V2 (CH01, PG9)        N160K                N7 glycan (197)

               120         *       140         *       160         *       180         *       200         *       220         *
HXB2       : DQSLKPCVKLTPLCVSLKCTDL--KNDTNTNSSSGRMIMEKGEIKNCSFNISTSIRGKVQKEYAFFYKLDIIPID-------NDTTSYKLTSCNTSVITQACPKVSFEPIPIHYC : 218
JR FL      : ...............T.N.K.V---.A...TNG.EGT-..R..........T....DE......L.....VV...-------.NN...R.I..D..........I.......... : 215
93A0HDC250 : .E.......I.....T.N...ARLQTNNS.T...PE.-------.......T.E..D.RK....L..RQ.V.Q.NNGIDKGTSNYSD.V.IN......K........Q....... : 219
08AO34HDP  : ...............T.N.NSIS-N.I.DS.DTVTSN--G.D.M......VT.ELKD.KK....L..R...VSLNKNSS---ENSSE.R.IN..S.TV.......N.D....... : 220
01PTHDECJN : ........Q......T.D.H.YS-..E..NTGMGED.---........Y..T.EL.D.K..V.SL..RP.VVKLNEA-----.NSST.R.IN...A.........T......... : 216
97PTINSA43 : ...............T.N...FG-NTT.KNT..NWET-.....M.......T....D.M.....L.....VV..EENKNNSSGNYSN.RMI..............T......... : 224
```

```
                                                              CD4bs (Loop D)    N280D           V3 loop  GPGR/Q motif   N332

                    240         *       260         *       280         *       300         *       320         *       340

HXB2       : APAGFAILKCNNKTFNGTGPCTNVSTVQCTHGIRPVVSTQLLLNGSLAEEEVVIRSVNFTDNAKTIIVQLNTSVEINCTRPNNNTRKRIRIQRGPGRAF-VTIGKIGNMRQAHCN : 332
JR FL      : ...........D.....K...K..................................D...N.........KE...............S.H.--......YT.GEI..DI...... : 328
93A0HDC250 : ...........D.K.....S.............K............I....II...K.ISN..........HT...E.I.LA.....S...--...QVLFA.GEI..DI...Y.. : 332
08AO34HDP  : ....Y................H...........K.................I...TKDLNVTQN....H.KEH...L..........SV..--...Q..YA.NAI..DI...... : 333
01PTHDECJN : ...........D.G.S.A.T.R.I.........K..A.............GI....E.I.N.........KEP.K...........RG.H.--....T.YAAGEI...I...... : 329
97PTINSA43 : ...........DNK.....................................I....E.....T.....H..E.I.......S...KRS.S.--......WT.GEVT.DI...... : 337


                                    CD4bs (VRC01-CD4 binding loop)                  V4 region                  CD4bs(VRC01-beta20/21)

                 *       360         *       380         *       400         *       420         *       440         *       460

HXB2       : ISRAKWNNNTLKQIASKLREQFGNNKTIIFKQSSGGDPEIVTHSFNCGGEFFYCNSTQLFNSTW-FNST-----WSTEGSNNTEGS------DTITLPCRIKQIINMWQKVGKAMY : 435
JR FL      : .......D.....VI......E-....V.NH.........M.......................-N.--------N.........-------N................E...... : 426
93A0HDC250 : .N.TS..S..HKVIG..K.H.K-....S.EPA....Q.....T............TSE...LSRL..GSGEETSNK.H----------------..........VR...R..Q.I. : 430
08AO34HDP  : .TEG...K..YEV.K..K.Y.P-.R..K.NS.....L.........A........TSL..E----R.V.ESNVTN.NS-T.S.TANNASISDKN......L.........A.R... : 442
01PTHDECJN : V.KTD..R..Q.V.IQ.K.H.T.ATR...NK.....L..T...............TSN....S.-E...M--TSNI.SE..-----------.....Q...........R..Q... : 430
97PTINSA43 : ..G.Q.....IH.VK..K.KL.-....V.N.........M.....R.................P....L--LSN..--W.S.K.I-----N..............L..E...... : 442
```

```
     G458Y  CD4bs (VRC01- beta23/24)  V5        end of gp120t        gp120/gp41 interface (PGT151)

                        *        480         *        500         *        520         *        540         *        560         *
HXB2        : APPISGQIRCSSNITGLLLTRDGGNSN--NESEIFRPGGGDMRDNWRSELYKYKVVKIEPLGVAPTKAKRRVVQREKRAVGIGALFLGFLGAAGSTMGAASMTLTVQARQLLSGI : 548
JR FL       : ....R...................INE--.GT..........K.........................................V........................L..... : 539
93A0HDC250  : ....A.N.T.T.............P..--GTN.T...T..................E...V.I..........E.......M..V................I.....V....... : 543
08AO34HDP   : ....E.N.I.R........V...TVN.----T.T...I....K.............E.K...I...G......E..........V................L...A....V.... : 553
01PTHDECJN  : ....P.V...E.............T..---G..T...E............................R......E..........V................I............. : 542
97PTINSA43  : ....R...N...K....I.........NS.GT.T......N.K..............L....I.....Q..........A.L--M................V.......L..... : 555

                                               N611A   gp120/gp41 interface (PGT151)

                   580         *        600         *        620         *        640         *        660         *        680         *
HXB2        : VQQQNNLLRAIEAQQHLLQLTVWGIKQLQARILAVERYLKDQQLLGIWGCSGKLICTTAVPWNASWSNKSLEQIWNHTTWMEWDREINNYTSLIHSLIEESQNQQEKNEQELLEL : 663
JR FL       : ...............RM..............V.......G...............................DR...NM.....E...D....E.YT................... : 654
93A0HDC250  : ....S...K.........K............V..........................T....S......Y.E..DNM..L..EK......GI.YN....A..T......D..A. : 658
08AO34HDP   : ....S........................T.VV.I............................S......Q.D...NM...Q.....S...YT.YQ.L...HI......KD..A. : 668
01PTHDECJN  : ....S.............K............V..L.A.....................T....S.....TYDS..GNM..LQ..K..S...YT.YD...K..........D..A. : 657
97PTINSA43  : ....K...N......................V..........................N....D.......DT..HNM.....E...D...D..YT.L.K............... : 670
```

W672A MPER (4E10/2F5)

```
              700         *        720         *        740         *        760         *        780         *        800

HXB2       : DKWASLWNWFNITNWLWYIKLFIMIVGGLVGLRIVFAVLSIVNRVRQGYSPLSFQTHLPTPRGPDRPEGIEEEGGERDRDRSIRLVNGSLALIWDDLRSLCLFSYHRLRDLLLIV : 778

JR FL      : ..........D..K.....I........I.....T..................L..A.....................G.....F.....V.................T. : 769

93A0HDC250 : ..........D.S......I........I....I....A.............L..LI.A.TEV...G....G...QG.T......S.F...A................FI..A : 773

08AO34HDP  : .S.N...S....S......I........I....I....A.I..............LT.N......LGR.......Q.....V...S.F.P.V..........C.RQ...CI... : 783

01PTHDECJN : ..........D..S.....RI..I.....I.........T.I..............LTHHQ.E.....R...G...Q.....V...S.F.T.V................FA..A : 772

97PTINSA43 : ..........D.SK.....I........I..........A..............RF.VR..............Q........AG.F.EI..V..........RL........ : 785


               *        820         *        840         *        860         *        880         *

HXB2       : TRIVELLGRR-------GWEALKYWWNLLQYWSQELKNSAVSLLNATAIAVAEGTDRVIEVVQGACRAIRHIPRRIRQGLERILL : 856

JR FL       : ..........-------...V.................................I..AL.RTY...L...T........A.. : 847

93A0HDC250 : A.T..T....-------...I...LG..AV..R.......I....T...V.......I..IA.RVF...I.........F..A.. : 851

08AO34HDP  : A.A......SSLRGLQR.......LKS.V...GL...K..IN..D.I..........I...I.R......N...T.L..F.AP.. : 868

01PTHDECJN : V.T.....HSSLKGLRL.......LK...S..G.......IN..DT......NW...I..IG.RTG...CN...........S.. : 857

97PTINSA43 : ..T..F....-------.......................................L.IL.R.F...L...T........A.. : 863
```

Figure S2- Amino acid sequence alignment of envelope sequences included in the present study and location of neutralizing epitopes.
Amino acid sequences of the envelope glycoproteins of isolates 93AOHDC250 (clade J), 08AO34HDP (clade C), 01PTHDECJN (clade CRF02_AG) and 97PTINSA43 (clade B) used to produce the immunogens of the study. The location of the neutralizing epitopes is indicated in relation to the reference HIV-1 strains HXB2 (KO3455) and JR-FL (AY669728) also included in the alignment. Location and name of the neutralizing epitopes and corresponding NAbs are shown in red. Amino acids corresponding to an epitope are shown in bold letters. Specific mutations that disrupt the neutralizing epitopes are shown in yellow. N7 glycan (position 197 in HXB2) is shown in purple. GPGR/Q motif is shown in light blue.

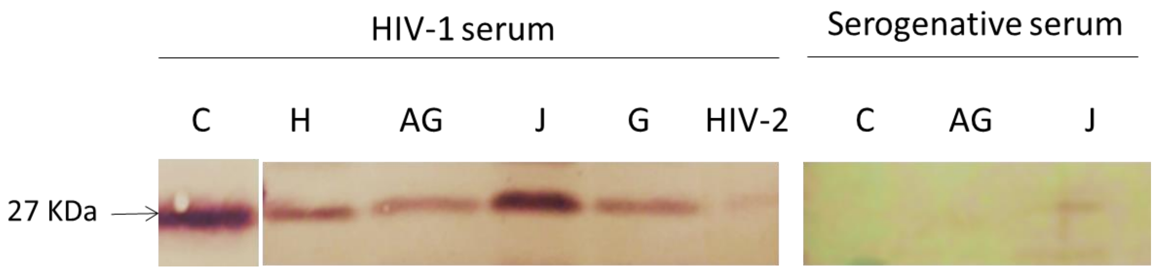

Figure S3 - Western-Blot analysis of the antigenic reactivity of C2V3C3 polypeptides. C2V3C3 polypeptides from HIV-1 clades C, H, CRF02_AG, J and G were expressed in Escherichia coli strain TOP10 and purified as indicated in Material and Methods. Recombinant polypeptides were incubated with sera from HIV-1 infected individuals (A) and with sera from healthy individuals (B). HIV-2 C2V3C3 polypeptide was used as positive control for this experiment because it has bout the same size of HIV-1 C2V3C3 and it shows some cross-reactivity with HIV-1 antiserum.

Group 3

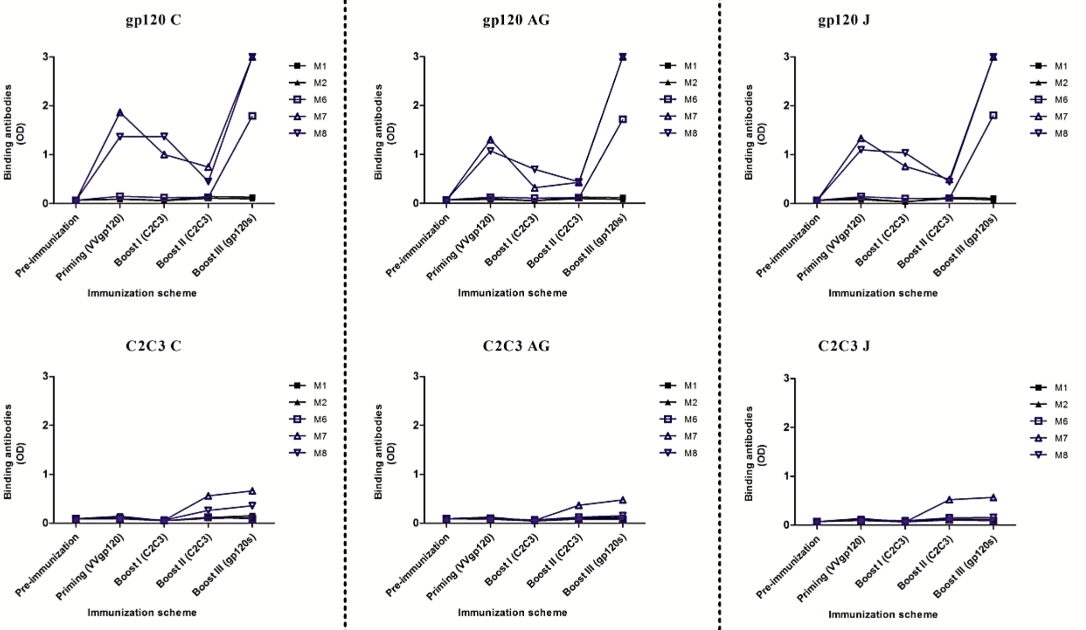

Group 4

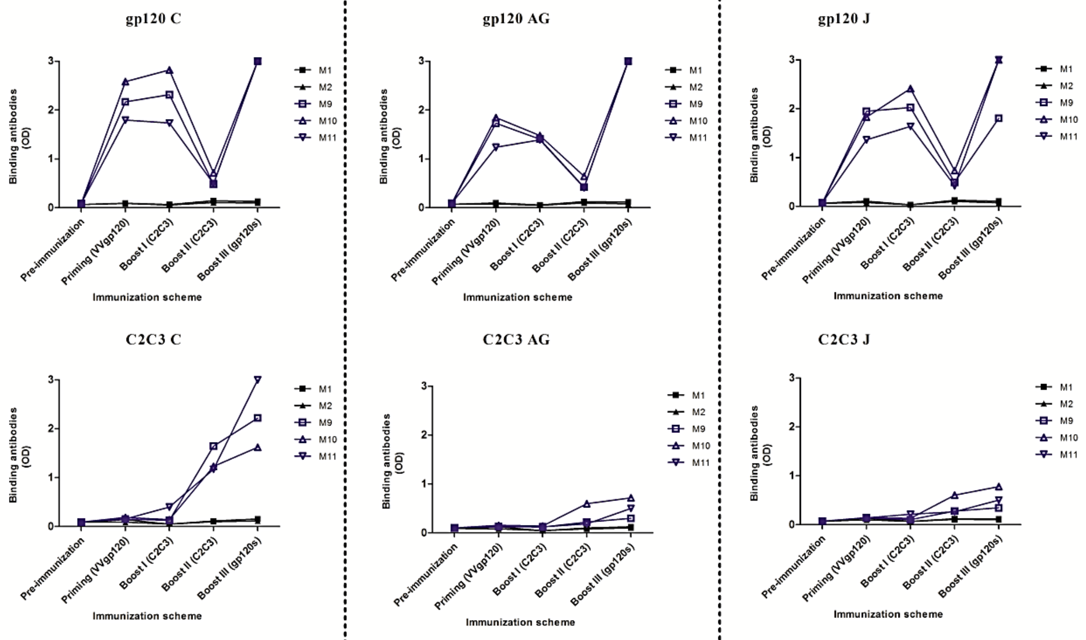

Group 5

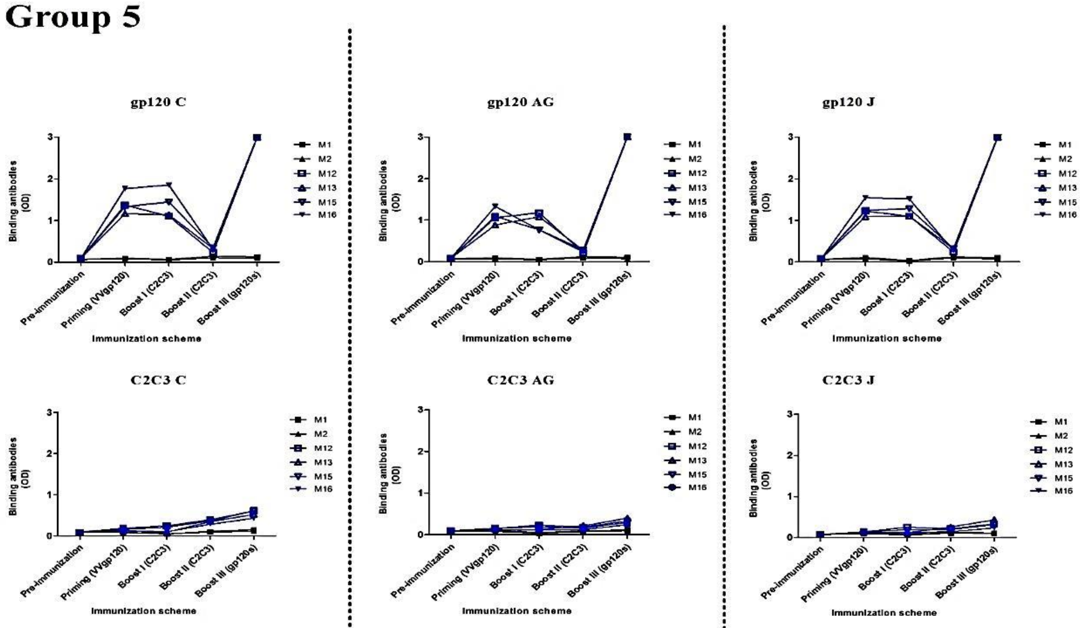

Group 6

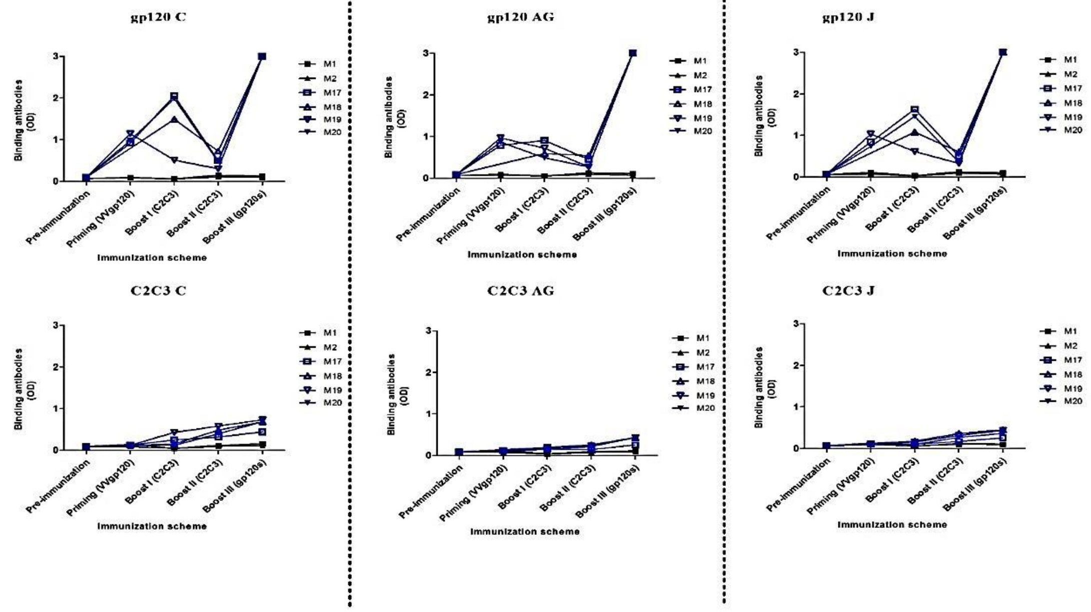

Figure S4 – Evolution of the binding antibody responses against Sgp120t glycoproteins and C2V3C3 polypeptides in BALB/c mice in the pilot study. Mice from groups 3 and 4 were primed with VV expressing gp120 and boosted with C2V3C3 polypeptides and Sgp120t from clade C. Mice from group 5 were primed VV expressing gp120 and boosted with C2V3C3 polypeptides and Sgp120t-AG; Mice from group 6 were primed with VV expressing gp120 and boosted with C2V3C3 polypeptides and Sgp120t-J. For all mice, the schedule of immunization included one priming and three boosts at days 15, 30 and 45. Fifteen days after each immunization, sera were collected and assayed for the presence of binding antibodies against HIV-1 immunogens. Blue lines represent immunized mice from the respective group; black lines represent mice from control group (G1-M1-M2).

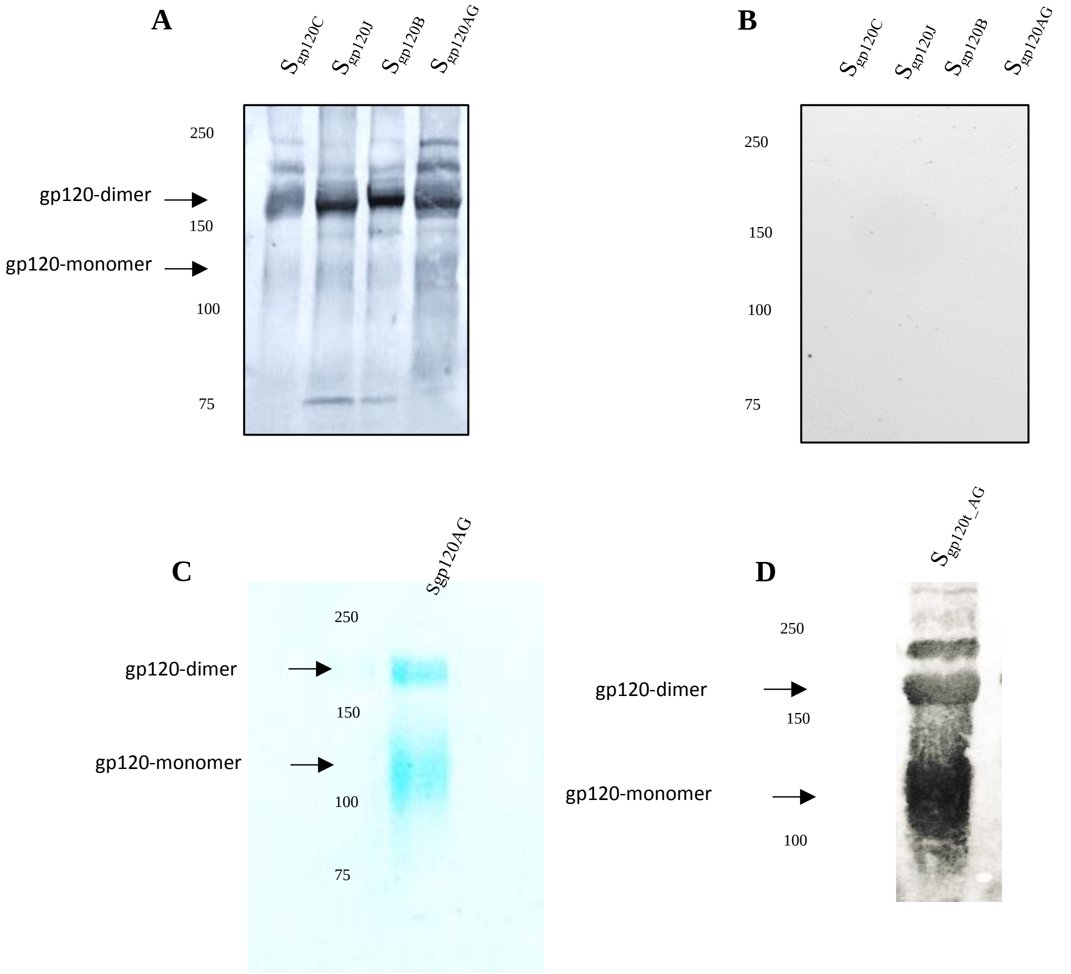

Figure S5- Western blot analysis of the reactivity of rabbits' serum with Sgp120t glycoproteins from different clades. A) Serum reactivity with Sgp120t from clades B, C, J and AG; B) Reactivity with serum from non-immunized rabbit; C) BlueSafe protein stain (Nzytech, Portugal) of gp120t-AG purified by lectin affinity chromatography analysed by 7.5% SDS-PAGE; D) Western-blot analysis of lectin affinity chromatography purified gp120t-AG using sera from immunized rabbit. Precision Plus Protein All Blue Standards (10-250 kDa) was used as a molecular weight marker.

Table S1- Characterization of the nine HIV-1 primary isolates included in the study

Sample	Year	Origin	Co-receptor use	*Env* subtype
93AOHDC249	1993	Angola	CCR5	U
93AOHDC250	1993	Angola	CCR5	J
93AOHDC251	1993	Angola	CCR5	H
93AOHDC252	1993	Angola	CCR5	U
93AOHDC253	1993	Angola	CCR5	J
08AO34HDP	2008	Angola	CCR5	C
01PTHDECJN	2001	Angola	CCR5	CRF02_AG
97PTINSA43	1997	Portugal	CCR5	B
00PTEBB	2000	Portugal	CCR5	G

Table S2- Primers used for polymerase chain reaction amplification of HIV-1 *Env*, C2V3C3 and truncated gp120.

Amplified fragment	Name	Orientation	Sequence (5′- 3′)[1]	Clade	HXB2 position
Full-length Env	PBENV1	Forward	CTATGGCAGGAAGAAGCGG	All	5968-5986
	PBENV2	Forward	CCACTGTCTTCTGCTCTTTC	All	6203-6223
	PBENV3	Reverse	AGTCATTGGTCTTARAGGTAC	All	9036-9016
	PBENV4	Reverse	TTTTGACCACTTGCCHCCCAT	All	8797A8817
Truncated gp120	RC1gp120*SalI*	Forward	TTGTGTGTCGACGAAAGAGCAGAAGAYAGTGGC	All	6203-6223
	RC2gp120BC*SalI*	Reverse	TTCTGTGTCGAC**CTA**ATATTTATATAATTCACTTCTC	B,C	7661-7682
	RC2gp120GAG*SalI*	Reverse	TTCTGTGTCGAC**CTA**ATACTTATATAATTCACTTCTC	G,CRF02_AG	7661-7682
	RC2gp120J*SalI*	Reverse	TTCTGTGTCGAC**CTA**ATATTTATATAACTCACTCCTC	J	7661-7682
	RC2gp120H*SalI*	Reverse	TTCTGTGTCGAC**CTA**ATATTTATATAGCTCACTTCTC	H	7661-7682
C2-V3-C3	HIV1EPIT11	Forward	TGTGGATCCCCAATTCCYATACATTATTG	All	6858-6878
	HIV1EPIT12	Reverse	TGAAAGCTTTCATCAGAAAAATTCYCCTCYAC	All	7374-7392

[1] Underlined letters indicate the restriction site for SalI; the stop codon **CTA is** indicated in bold letters.

Table S3- Primers used for sequencing of HIV-1 *env* gene segments

Primer	Orientation	Sequence (5´- 3´)	HXB2 position
PBSEQ1	Forward	AGCYTAAAGCCATGTGT	6567 – 6583
PBSEQ2	Reverse	ACACATGGCTTTARGCT	6567 – 6583
PBSEQ3	Forward	CAGTACAATGTACACA	6955 – 6970
PBSEQ4	Reverse	TGTGTACATTGTACTG	6955 – 6970
PBSEQ5	Forward	CATAGTTTTAATTGTRGAGG	7344 – 7363
PBSEQ6	Reverse	CCTCYACAATTAAAACTATG	7344 – 7363
PBSEQ13	Forward	GGACAATTGGAGAAGTGAA	7652 – 7670
PBSEQ7	Forward	GAGAGAAAAAAGAGCAGT	7745 – 7762
PBSEQ8	Reverse	ACTGCTCTTTTTTCTCTC	7745 – 7762
PBSEQ9	Forward	ATCTGCACCACTAATGT	8031 – 8047
PBSEQ10	Reverse	ACATTAGTGGTGCAGAT	8031 – 8047
PBSEQ11	Forward	CCTGTGCCTCTTCAGCTACC	8510 – 8529
PBSEQ12	Reverse	GGTAGCTGAAGAGGCACAGG	8510 – 8529

Table S4- Characteristics of the global panel of twelve tier 2 HIV-1 Env-pseudoviruses used in neutralization assays.

Env- pseudoviruses	HIV-1 Clade	Origin	Year
TRO11	B	Italy	1995
25710	C	India	1999
398F1	A	Tanzania	2001
X2278	B	Spain	2007
BJOX2000	CRF07_BC	China	2007
X1632	G	Spain	2004
CE1176	C	Malawi	2004
246F3	AC recomb	Tanzania	2001
CH119	CRF07_BC	China	2004
CE0217	C	Malawi	2007
CNE55	CRF01_AE	China	2007
CNE8	CRF01_AE	China	2006

Table S5- Baseline data of the neutralization assays performed in mice and rabbits

Viruses	Strains (pseudoviruses and primary isolates)[1]	Relative luminescence units [mean, of 3-5 measurements (standard deviation)]					
		Mice (main study)			Rabbits		
		Virus only	Virus + Pre immune sera	% Baseline Neutralization	Virus only	Virus + Pre immune sera	% Baseline Neutralization
**HIV-1 (Tier 1 neutralization)**	NL4.3	nd	nd	-	49,772 (1,941)	61,596 (1,897)	-23
	SG3.1	43,523 (1,700)	68,037 (3,164)	-56	52,873 (3,712)	76,596 (7,270)	-45
**HIV-1 (Tier 2 neutralization)**	PCNE8	nd	nd	-	19,858 (673)	16,953 (719)	15
	PX2278	86,303 (4,569)	57,898 (1,630)	33	48,525 (4,538)	42,368 (1,737)	13
	TRO11	42,267 (1,827)	53,496 (4,414)	-27	89,112 (10,972)	107,804 (23,459)	-21
	PCH119	105,465 (3,693)	141,512 (9,788)	-34	108,874 (1,981)	130,004 (30,612)	-19
	CNE55	39,115 (2,552)	68,500 (6,939)	-75	34,951 (1,876)	45,604 (2,688)	-30
	398F1	22,453 (2,089)	18,073 (2,185)	20	43,156 (3,361)	37,602 (821)	13
	X1632	48,372 (3,504)	88,902 (7,736)	-84	34,740 (3,778)	46,808 (2,275)	-35
	PCE1176	17,543 (1,253)	12,943 (932)	26	59,491 (3,091)	71,267 (2,091)	-20
	25710243	46,636 (4,047)	46,355 (2,068)	1	125,840 (6,453)	158,903 (24,945)	-26
	PCE0217	71,450 (7,997)	146,026 (10,781)	-104	104,005 (7,239)	120,531 (674)	-16
	BJOX2000	27,445 (3,300)	39,308 (5,198)	-43	73,659 (11,352)	113,412 (21,529)	-54
	246F3	25,363 (3,224)	46,895 (1,863)	-85	49,245 (9,551)	68,287 (7,031)	-39
	93HDC253*	19,141 (5,025)	22,644 (2,598)	-18	20,637 (416)	19591 (187)	5
	01PTCJN*	32,035 (2,863)	84,374 (3,581)	-163	52,518 (4,809)	49,207 (6,754)	6
	93HDC250*	427,353 (45,718)	487,798 (26,556)	-14	nd	nd	-
	93AOHDC249*	nd	nd	-	63,077 (12,006)	65,420 (756)	-4
	93AOHDC252*	nd	nd	-	43,723 (2,192)	41,160 (2,464)	6
**HIV-2**	HCC19.03*	nd	nd	-	46,119 (4,595)	43,684 (2,468)	5
	AUC*	nd	nd	-	44,255 (3,737)	40,973 (2,683)	9

**VSV**	-	77,943 (8,718)	68,468 (8,056)	12	28,651 (2,453)	28,170 (440)	2

[1]Primary isolates are indicated with an asterisk; nd- not one.
